# Supplementary material for: The value of inquiring about functional impairments for early identification of inflammatory arthritis: a large cross-sectional derivation and validation study from the Netherlands
Source: BMJ Open. 2020 Dec 13;10(12):e040148. doi: 10.1136/bmjopen-2020-040148 (PMC7737110; doi:10.1136/bmjopen-2020-040148)
Supplement: Supplementary data [file bmjopen-2020-040148supp001.pdf]

Supplementary file

Table S1. Discriminative ability for the presence of IA of the total HAQ-score and its individual questions in the derivation cohort (n = 997)

Category	Item[*]	AUC (95%CI)	p
–	Total HAQ-score	0.55 (0.52–0.59)	0.006
**(1) Dressing & grooming**	(A) … Dress yourself, including shoelaces and buttons?	0.58 (0.55–0.62)	<0.001
	(B) … Wash your hair?	0.52 (0.48–0.56)	0.301
**(2) Arising**	(A) … Stand up from a straight chair?	0.55 (0.51–0.58)	0.016
	(B) … Get in and out of bed?	0.53 (0.49–0.57)	0.104
**(3) Eating**	(A) … Cut meat?	0.50 (0.46–0.54)	0.897
	(B) … Lift a full cup or glass to your mouth?	0.51 (0.47–0.55)	0.619
	(C) … Open a new milk carton?	0.48 (0.45–0.52)	0.372
**(4) Walking**	(A) … Walk outdoors on flat ground?	0.57 (0.53–0.60)	0.001
	(B) … Climb up five steps?	0.56 (0.52–0.59)	0.003
**(5) Hygiene**	(A) … Wash and dry your body?	0.54 (0.50–0.58)	0.041
	(B) … Get in and out of a tub bath?	0.53 (0.49–0.57)	0.138
	(C) … Get on and off the toilet?	0.56 (0.52–0.59)	0.003
**(6) Reach**	(A) … Reach and get down a 2½kg object (such as a bag of sugar) from above your head?	0.51 (0.47–0.55)	0.567
	(B) … Bend down to pick up clothing from the floor?	0.52 (0.48–0.56)	0.300
**(7) Grip**	(A) … Open car doors?	0.53 (0.49–0.57)	0.145
	(B) … Open previously opened jars?	0.49 (0.45–0.52)	0.464
	(C) … Turn a faucet on and off?	0.51 (0.47–0.55)	0.514
**(8) Activities**	(A) … Run errands and shop?	0.55 (0.51–0.59)	0.007
	(B) … Get in and out of a car?	0.54 (0.50-0.58)	0.037
	(C) … Do chores such as vacuuming or yard work?	0.55 (0.51–0.59)	0.011

Legend

* Names of categories are shown, as well as individual questions (at the EA*R*C, Dutch translations were used). Each question starts with 'Are you able to …'. Patients indicated the degree of difficulties on each question with 'without any difficulty' (0), 'with some difficulty' (1), 'with much difficulty' (2) or 'unable to do' (3).

Abbreviations: IA = inflammatory arthritis; HAQ = Health Assessment Questionnaire Disability Index; AUC = area under the receiver operating characteristics curve; CI = confidence interval.

Table S2. Presence of difficulties with dressing associated with presence of IA independently from other clinical variables* (detailed overview of multivariable model)

		Derivation cohort (n = 997) OR (95%CI)	1st Validation cohort (n = 506) OR (95%CI)	2nd Validation cohort (n = 557) OR (95%CI)
**Male**		1.99 (1.47–2.70)	1.36 (0.91–2.03)	2.47 (1.66–3.68)
**Age ≥ 60 years**		1.82 (1.35–2.45)	1.40 (0.93–2.10)	1.90 (1.28–2.83)
**Symptom duration in weeks**	≥ 52	1.00 (ref)	1.00 (ref)	1.00 (ref)
	0–5.99	3.63 (2.45–5.39)	1.48 (0.86–2.53)	2.39 (1.38–4.17)
	6–51.99	2.16 (1.50–3.13)	1.24 (0.77–1.99)	1.92 (1.13–3.27)
**Acute onset of symptoms**		0.97 (0.72–1.32)	1.05 (0.69–1.58)	1.19 (0.77–1.83)
**Morning stiffness > 60 minutes**		1.34 (0.84–2.15)	1.59 (0.86–2.93)	0.84 (0.45–1.57)
**Number of painful joints**	0	1.00 (ref)	0.00**	1.00 (ref)
	1–3	4.45 (1.36–14.53)	1.54 (0.95–2.50)	2.70 (0.66–11.00)
	≥ 4	2.34 (0.73–7.53)	1.00 (ref)	1.60 (0.40–6.44)
**Patient-reported swollen joint(s) ≥ 1**		1.97 (1.27–3.04)	1.76 (0.95–3.25)	1.74 (1.07–2.81)
**Difficulties with making a fist**		1.31 (0.92–1.88)	1.54 (0.94–2.54)	1.57 (1.01–2.46)
**Difficulties with dressing**	yes (≥ 1)	1.71 (1.27–2.32)	1.64 (1.08–2.50)	1.87 (1.20–2.92)

Legend

Results were pooled across 30 imputations.

* These variables were selected based on a recent study from the Leiden EA*R*C that determined clinical variables that associated with the presence of IA.

** In the Groningen validation cohort none of the patients who indicated no painful joints on the questionnaire were positive for IA upon joint examination. Therefore, no meaningful CI could be calculated for this group and patients with ≥4 painful joints were assigned as reference group instead.

Abbreviations: IA = inflammatory arthritis; OR = odds ratio; CI = confidence interval, ref = reference.

Table S3. Characteristics of patients with and without available data on difficulties with dressing

	Derivation cohort			1st Validation cohort			2nd Validation cohort		
**Data on difficulties with dressing:**	**Available (n = 956)**	**Missing (n = 41)**	**p***	**Available (n = 495)**	**Missing (n = 11)**	**p***	**Available (n = 542)**	**Missing (n = 15)**	**p***
**Male, n (%)**	275 (29)	15 (37)	0.294	174 (35)	3 (27)	0.755	180 (33)	3 (20)	0.406
**Age in years, mean ± SD**	51 ± 16	57 ± 17	0.015	51 ± 16	57 ± 10	0.237	54 ± 16	58 ± 12	0.312
**Symptom duration in weeks, median (IQR)**	13 (4–62)	9 (3–72)	0.792	18 (5–69)	7 (5–64)	0.519	13 (4–59)	13 (5–24)	0.750
**Acute onset of symptoms, n (%)**	353 (38)	15 (41)	0.731	185 (38)	2 (20)	0.333	201 (37)	2 (29)	0.713
**Morning stiffness in minutes, median (IQR)**	10 (0–30)	4 (0–60)	0.983	10 (0–33)	10 (1–190)	0.776	10 (0–30)	0 (0–1)	0.012
**Number of painful joints, median (IQR)**	7 (3–14)	6 (2–25)	0.941	9 (4–18)	9 (3–20)	0.840	8 (3–17)	1 (1–9)	0.021
**Number of patient-reported swollen joints, median (IQR)**	2 (1–5)	2 (0–5)	0.503	3 (1–8)	5 (2–8)	0.698	2 (1–5)	1 (0–5)	0.317
**Difficulties with making a fist, n (%)**	239 (48)	3 (23)	0.095	128 (54)	2 (50)	1.000	288 (55)	3 (50)	1.000

Legend

Percentages are based on available questionnaire entries.

* Unpaired t-tests, Fisher's Exact tests and Mann-Whitney U tests were used as appropriate.

Abbreviations: SD = standard deviation; IQR = interquartile range.

Table S4. (sensitivity analyses limited to unimputed data) Association between presence of difficulties with dressing and presence of IA in the derivation and validation cohorts

	OR (95%CI) univariable	OR (95%CI) age, gender adjusted	OR (95%CI) multivariable*
**Derivation cohort**	1.83 (1.41–2.38)	1.87 (1.43–2.44)	1.59 (0.98–2.57)
**1st Validation cohort**	2.02 (1.41–2.91)	1.92 (1.33–2.77)	1.70 (0.83–3.84)
**2nd Validation cohort**	2.19 (1.51–3.17)	1.99 (1.36–2.93)	1.91 (1.16–3.16)

Legend

* Point estimates are similar to those obtained in the main analyses, but have broader confidence intervals since only complete cases could be analysed. Covariates: being male, age ≥60 years, symptom duration, acuteness of symptom onset, morning stiffness >60 minutes, number of painful joints, presence of patient-reported swollen joint(s) and difficulties with making a fist.

Abbreviations: IA = inflammatory arthritis; OR = odds ratio; CI = confidence interval.
