# Supplementary material for: The value of inquiring about functional impairments for early identification of inflammatory arthritis: a large cross-sectional derivation and validation study from the Netherlands
Source: BMJ Open. 2020 Dec 13;10(12):e040148. doi: 10.1136/bmjopen-2020-040148 (PMC7737110; doi:10.1136/bmjopen-2020-040148)
Supplement: Author's manuscript [file bmjopen-2020-040148.draft_revisions.pdf]

BMJ Open is committed to open peer review. As part of this commitment we make the peer review history of every article we publish publicly available.

When an article is published we post the peer reviewers' comments and the authors' responses online. We also post the versions of the paper that were used during peer review. These are the versions that the peer review comments apply to.

The versions of the paper that follow are the versions that were submitted during the peer review process. They are not the versions of record or the final published versions. They should not be cited or distributed as the published version of this manuscript.

BMJ Open is an open access journal and the full, final, typeset and author-corrected version of record of the manuscript is available on our site with no access controls, subscription charges or pay-per-view fees (http://bmjopen.bmj.com).

If you have any questions on BMJ Open's open peer review process please email info.bmjopen@bmj.com

BMJ Open

The value of inquiring about functional impairments for early identification of inflammatory arthritis: a large cross-sectional derivation and validation study

Journal:	*BMJ Open*
Manuscript ID	bmjopen-2020-040148
Article Type:	Original research
Date Submitted by the Author:	06-May-2020

Complete List of Authors:	van Dijk, Bastiaan; Leiden University Medical Center,<br>van Steenbergen, Hanna<br>Niemantsverdriet, Ellis; Leiden University Medical Center, Rheumatology<br>Brouwer, Elisabeth; University Medical Centre Groningen, Rheumatology and Clinical Immunology<br>van der Helm-van Mil, Annette; Leiden University Medical Center, Rheumatology; Erasmus Medical Center, Rheumatology

Keywords:	RHEUMATOLOGY, PRIMARY CARE, Rheumatology < INTERNAL MEDICINE

SCHOLARONE™
Manuscripts

I, the Submitting Author has the right to grant and does grant on behalf of all authors of the Work (as defined in the below author licence), an exclusive licence and/or a non-exclusive licence for contributions from authors who are: i) UK Crown employees; ii) where BMJ has agreed a CC-BY licence shall apply, and/or iii) in accordance with the terms applicable for US Federal Government officers or employees acting as part of their official duties; on a worldwide, perpetual, irrevocable, royalty-free basis to BMJ Publishing Group Ltd ("BMJ") its licensees and where the relevant Journal is co-owned by BMJ to the co-owners of the Journal, to publish the Work in this journal and any other BMJ products and to exploit all rights, as set out in our licence.

The Submitting Author accepts and understands that any supply made under these terms is made by BMJ to the Submitting Author unless you are acting as an employee on behalf of your employer or a postgraduate student of an affiliated institution which is paying any applicable article publishing charge ("APC") for Open Access articles. Where the Submitting Author wishes to make the Work available on an Open Access basis (and intends to pay the relevant APC), the terms of reuse of such Open Access shall be governed by a Creative Commons licence – details of these licences and which Creative Commons licence will apply to this Work are set out in our licence referred to above.

Other than as permitted in any relevant BMJ Author's Self Archiving Policies, I confirm this Work has not been accepted for publication elsewhere, is not being considered for publication elsewhere and does not duplicate material already published. I confirm all authors consent to publication of this Work and authorise the granting of this licence.

For peer review only

Original research

The value of inquiring about functional impairments for early identification of inflammatory arthritis: a large cross-sectional derivation and validation study

Bastiaan T. van Dijk[1], Hanna W. van Steenbergen[1], Ellis Niemantsverdriet[1], Elisabeth Brouwer[2], Annette H.M. van der Helm-van Mil[1,3]

[1] Department of Rheumatology, Leiden University Medical Centre, Leiden, the Netherlands

[2] Department of Rheumatology and Clinical Immunology, University Medical Centre Groningen, Groningen, the Netherlands

[3] Department of Rheumatology, Erasmus Medical Centre, Rotterdam, the Netherlands

Corresponding author

B.T. van Dijk, M.D., Department of Rheumatology, Leiden University Medical Centre

P.O. Box 9600, 2300 RC Leiden, the Netherlands

Tel.: +31 71 5265655; Email: B.T.van_Dijk@lumc.nl

ORCID ID: 0000-0002-5161-6791

Word count (main text): 3097

For peer review only - http://bmjopen.bmj.com/site/about/guidelines.xhtml

ABSTRACT

Objectives. Healthcare professionals other than rheumatologists experience difficulties in detecting early inflammatory arthritis (IA) by joint examination. Self-reported symptoms are increasingly considered as helpful and could be incorporated in online tools to assist healthcare professionals, but firstly their discriminative ability must be assessed. As part of this effort, we evaluated whether inquiring about functional impairments could aid early IA-identification.

Design. Cross-sectional derivation and validation study.

Setting. Data from two Early Arthritis *Recognition* Clinics (EA*R*C) in the Netherlands were studied, which are easy-access outpatient rheumatology clinics intermediary between primary and secondary care for patients in whom GPs suspect but are unsure about IA-presence.

Participants. Between 2010-2014, 997 patients consecutively visited the Leiden-EA*R*C (derivation cohort). Patients consecutively visiting the Groningen-EA*R*C (2010-2014, n=506) and Leiden-EA*R*C (2015-2018, n=557) served as validation cohorts.

Primary and secondary outcome measures. Physical functioning was assessed with the Health Assessment Questionnaire Disability-Index (HAQ); IA-presence by physical joint examination by rheumatologists. HAQ-questions were studied individually regarding discriminative ability for IA-presence. For the best discriminating question, odds ratios (ORs) and positive predictive values (PPVs) for IA-presence were determined.

Results. IA was ascertained in 43% (derivation cohort), 53% and 35% (validation cohorts). In the derivation cohort, IA-presence associated with higher mean HAQ-scores (0.84 versus 0.73, p=0.003). One question on difficulties with dressing equalled discriminative ability of the total HAQ-score. 'Difficulties with dressing' yielded ORs for IA-presence of 1.8(95%CI

For peer review only - http://bmjopen.bmj.com/site/about/guidelines.xhtml

1.4-2.4) in the derivation cohort; 2.0(1.4-2.9) and 2.1(1.5-3.1) in the validation cohorts. After adjustments for clinical characteristics these were 1.7(1.3-2.3), 1.6(1.1-2.5) and 1.9(1.2-2.9). PPVs were 50%, 60% and 42%, respectively.

Conclusions. Patient-reported difficulties with dressing in patients with suspected IA associated with actual IA-presence. Although further validation is required, e.g. in primary care, this simple question could be of help in future early IA-detection tools for healthcare professionals with limited experience in joint examination.

For peer review only

For peer review only - http://bmjopen.bmj.com/site/about/guidelines.xhtml

STRENGHTS AND LIMITATIONS OF THIS STUDY

- We evaluated the value of questions about functional impairments for early recognition of inflammatory arthritis (IA), for which new and simple tools are urgently needed – especially in primary care.
- This was studied in a setting intermediary between primary and secondary care that services patients whose general practitioners (GPs) were doubtful about the presence of IA; the population for whom such new tools are most relevant.
- One simple question about difficulties with dressing was associated with presence of IA, which equalled discriminative ability of the total HAQ but is more suitable for busy daily practice.
- This association was tested for independence from known predictors and validated in two independent cohorts.
- However, further validation in primary care is needed, since data were not yet collected in actual primary care practices.

For peer review only

For peer review only - http://bmjopen.bmj.com/site/about/guidelines.xhtml

INTRODUCTION

In rheumatoid arthritis (RA), early initiation of treatment with disease-modifying antirheumatic drugs (DMARDs) is associated with improved outcomes.[1,2] Also for other types of inflammatory arthritis (IA) the importance of early treatment is increasingly recognised.[3-5] To achieve early treatment initiation however, it is necessary for at-risk patients to be referred to a rheumatologist in a timely fashion.[6]

Consequently, general practitioners (GPs) play a crucial role in early identification of IA, but they have indicated considerable barriers regarding timely and adequate referral of patients with musculoskeletal complaints, including a lack of self-confidence in detecting synovitis.[7] GPs generally have limited experience with the detection of IA by joint palpation compared to rheumatologists, since their patient population is characterised by a relatively low incidence of RA and IA (0.7/1000 patient years in the Netherlands, which amounts to 1–2 new patients on average per GP per year) compared to a much higher incidence of other musculoskeletal complaints.[8-10] These circumstances may add to referral delay at the level of the initial healthcare provider (usually the GP), which was found to be an important contributor to overall delay until treatment initiation in many European countries.[11]

In this light, the search for novel strategies to improve early recognition and referral of patients at risk for IA and RA remains of great importance. This was emphasised in the latest update of the European League against Rheumatism (EULAR) research agenda for early arthritis by including 'the identification of tools that could help GPs diagnose early arthritis and prioritise referral.'[6] Given the increasing usage of healthcare-related mobile applications (apps)[12], new tools are preferred to be simple and time-efficient, providing suitability for possible future digital initiatives for early IA-screening. In addition to ease-of-

For peer review only - http://bmjopen.bmj.com/site/about/guidelines.xhtml

use, such tools should be based on valid and reliable data but currently most medical mobile apps are not properly evidence-based, highlighting the need for thorough research.[13]

Patients with IA generally experience limitations in physical functioning. One could hypothesise that these limitations may differ in sort and severity compared to those related to other, less urgent causes of joint symptoms. Functional impairments in patients with rheumatic diseases are generally assessed using the Health Assessment Questionnaire Disability-Index (HAQ),[14] but use of this patient-reported outcome instrument is mostly limited to research in established rheumatic disease instead of earlier stages. Nonetheless, it is generally considered a self-explanatory and relatively efficient questionnaire (it can be completed within five minutes).[15] In order to search for tools that might facilitate early identification of IA we performed a cross-sectional study on the association between functional impairments (measured by the HAQ) and presence of IA at joint examination (identified by rheumatologists). Aiming for simple and time-efficient use in daily practice, we also studied the HAQ-questions separately and evaluated whether a single question could facilitate early identification of IA. Although information on functional impairments alone is not expected to have sufficient accuracy, it may be used as part of a more comprehensive tool that could be incorporated in a screening app for early IA. Findings were verified in two validation cohorts.

PATIENTS AND METHODS

Patients

Aiming to decrease referral delay in early IA, Early Arthritis *Recognition* Clinics (EA*R*C) were initiated in 2010 at the Leiden and Groningen University Medical Centers (LUMC and UMCG) in the Netherlands. The EA*R*C design has been described previously.[16] In short,

For peer review only - http://bmjopen.bmj.com/site/about/guidelines.xhtml

EA*R*Cs are clinics for patients in whom their GP suspects but is unsure about the presence of IA. These easy-access clinics are characterised by their unique intermediate setting, namely in between primary and secondary care. The EA*R*Cs have no waiting lists, can be visited by referred patients once to twice a week without an appointment and have markedly reduced referral delay.[16] GPs in the region are instructed to quickly refer any patient for whom they are unsure about the presence of IA, instead of applying a strategy of 'watchful waiting' or performing additional diagnostic tests. Notably, auto-antibodies or other additional investigations are generally not performed by GPs in our region[17], which is in accordance with the Dutch national guidelines.[18]

At the EA*R*C, patients completed a brief clinical questionnaire on their symptoms, followed by a short consultation and full 66-joint examination by an experienced rheumatologist. If IA was detected at physical examination (by palpable joint swelling), patients were analysed at the regular rheumatology outpatient clinic for further diagnosis. Otherwise, they were referred back to their GP. Laboratory tests and imaging were not performed at the EA*R*C.

The derivation cohort comprised 997 patients who consecutively visited the Leiden-EA*R*C between September 2010 and April 2014. The first validation cohort consisted of 506 patients who visited the Groningen-EA*R*C from September 2010 to January 2014. The second validation cohort consisted of 557 patients who visited the Leiden-EA*R*C from November 2015 to December 2018. Patients who visited the Leiden-EA*R*C from April 2014 to November 2015 did not fill out questions on physical functioning and were not included in this study.

For peer review only - http://bmjopen.bmj.com/site/about/guidelines.xhtml

Patient involvement

Patient research partners agreed with the pathway of care at EA*R*C and provided feedback on the questionnaire, which was expanded in 2012 with two questions.

HAQ

Physical functioning was assessed during the same EA*R*C-visit using the HAQ, a questionnaire containing 20 questions on impairments across eight functional categories.[14,15] Each question was scored by patients on a 4-point scale ranging 0–3, representing the degree of difficulties experienced when performing the corresponding activity, with 0 indicating no difficulties and 3 indicating full disability. The total HAQ score is calculated as the average across eight categories and ranges 0–3. Patients in the derivation cohort and patients in the first validation cohort filled out the full HAQ. Based on initial results from the derivation cohort, patients in the second validation cohort were asked to complete only question 1A on difficulties with dressing.

Outcome

The outcome used in all statistical analyses was presence of IA, determined by the rheumatologist at physical examination. Final classifying diagnoses were made during subsequent visit(s) at the regular rheumatology outpatient clinic and were beyond the scope of this study.

For peer review only - http://bmjopen.bmj.com/site/about/guidelines.xhtml

Statistical analyses

In the derivation cohort, total HAQ-scores were compared between patients with and without IA at joint examination. Areas under the curve (AUCs) were determined for the total HAQ-score as well as its questions individually, in order to select the best discriminating question for further investigation (based on highest AUC). The use of aids and devices was taken into account for calculation of the total HAQ-score as prescribed, but not for scores on individual questions.[14,15] For the best discriminating question, odds ratios (ORs) and AUCs were determined with both the original, categorical responses and with binary responses (no vs. any degree of difficulties; i.e. scores ≥1), since a binary tool would be even more suited for easy use in daily practice. ORs were adjusted for age and gender.[19-21] Test characteristics were calculated. Analyses were repeated in the two validation cohorts.

A previous study from the Leiden-EA*R*C derived an algorithm for IA-identification, consisting of clinical variables that were independently associated with the presence of IA (being male, age ≥60 years, symptom duration, acuteness of symptom onset, morning stiffness >60 minutes, number of painful joints, presence of patient-reported swollen joint(s) and difficulties with making a fist).[21] These variables were included in multivariable logistic regression analyses, to study if self-reported functional disabilities were independently associated with IA. In addition, change in diagnostic accuracy when adding functional disability to the algorithm was assessed in the derivation cohort by comparing AUCs.

The HAQ-question of interest was missing in 4%, 2% and 3% of the cohorts respectively. Characteristics of patients with available and missing data on this HAQ-question were compared. Missing values were imputed using multiple imputation by chained equations (MICE) with predictive mean matching (PMM).[22] Rubin's rules were applied to pool point estimates and confidence intervals across 30 imputations.[23] Imputed data were used for the

For peer review only - http://bmjopen.bmj.com/site/about/guidelines.xhtml

main analyses. In sensitivity analyses, results were compared to those obtained from the unimputed data.

IBM SPSS (version 25) and R (version 3.5.2) were used. P-values <0.05 were considered statistically significant.

RESULTS

Patients

Patient characteristics for all three cohorts are presented in Table 1. Over 80% in each cohort presented with hand and/or wrist arthralgia. IA was diagnosed in 43%, 53% and 35% of patients, respectively. Patients in the first validation cohort had a slightly longer symptom duration (median 18 weeks) than patients in the derivation and second validation cohorts (median 12 and 13 weeks, respectively). All other characteristics were similar.

Discriminative ability of a single question equalled the total HAQ score

Patients with IA at physical examination had a higher mean total HAQ score than patients without IA in the derivation cohort (0.84 (95%CI 0.77–0.89) compared to 0.73 (0.67–0.77), p=0.003), indicating a higher degree of functional disability. To search for a method that is simple and time-efficient for usage in daily practice, the HAQ-questions were studied individually (supplementary Table S1). Of these questions, scores on 'difficulties with dressing' (question 1A) yielded the highest AUC, which equalled the AUC of the total HAQ score: 0.58 (0.55–0.62) vs. 0.55 (0.52–0.59), respectively. This question ("Are you able to dress yourself, including shoelaces and buttons?") was therefore considered the best discriminating question and was studied further. The degree of difficulties with dressing associated with the presence of IA in the derivation cohort (Table 2). However, because loss

For peer review only - http://bmjopen.bmj.com/site/about/guidelines.xhtml

of overall discriminative ability induced by dichotomisation into a binary score was minor (AUC of 0.57 (0.54–0.61) vs. 0.58 (0.55–0.62)) and a binary question is easier to implement in practice, a binary variable was used in subsequent analyses.

Difficulties with dressing were independently associated with presence of IA in derivation cohort

ORs for the IA-presence of a confirmative answer on the question about difficulties with dressing are presented in Table 3. In the derivation cohort, the presence of difficulties with dressing (scores ≥1) was associated with presence of IA with an age and gender adjusted OR of 1.83 (1.41–2.39). After further adjustment for the aforementioned clinical variables that were previously identified as associated with IA (being male, age ≥60 years, symptom duration, acuteness of symptom onset, morning stiffness >60 minutes, number of painful joints, presence of patient-reported swollen joint(s) and difficulties with making a fist), difficulties with dressing remained independently associated with the presence of IA, with an OR of 1.71 (1.27–2.32). ORs for the other clinical variables are provided in the supplementary file (supplementary Table S2). The AUC of the model with only the aforementioned clinical variables was 0.72 (0.69–0.76), while the model where difficulties with dressing was added had an AUC of 0.73 (0.70–0.77). Since the presence of difficulties with dressing was independently associated with IA it was subsequently studied in the validation cohorts.

For peer review only - http://bmjopen.bmj.com/site/about/guidelines.xhtml

Difficulties with dressing were independently associated with presence of IA in both validation cohorts

In the first and second validation cohorts, age and gender adjusted ORs were 1.89 (1.31–2.72) and 1.95 (1.32–2.86), respectively. After further adjustment for the aforementioned clinical variables that were previously identified as associated with IA, ORs were 1.64 (1.08–2.50) and 1.87 (1.20–2.92), respectively. Thus, the independent association of difficulties with dressing with presence of IA was conformed in both validation cohorts.

Predictive values and test characteristics

Lastly, we studied test characteristics and predictive values of positive answers on just the single question about difficulties with dressing (Figure 1). The negative predictive values (NPVs) in the different cohorts ranged between 57–74%, indicating that 57-74% of patients without difficulties with dressing had no IA at physical examination. The positive predictive values (PPVs) ranged between 42–60%, meaning that 42–60% of patients who reported to have difficulties with dressing were indeed identified as having IA.

Sensitivity analyses on unimputed data

No clinically relevant differences in characteristics of patients with and without information on 'difficulties with dressing' were found (supplementary Table S3). Sub-analyses limited to the unimputed data (supplementary Table S4) yielded similar results as in the main analyses.

For peer review only - http://bmjopen.bmj.com/site/about/guidelines.xhtml

For peer review only

DISCUSSION

Healthcare professionals who are not rheumatologists generally have limited experience in joint examination, which hampers them in early identification of IA. For GPs this contributes to referral delay and necessitates the development of feasible tools that could support early identification of IA. We investigated the value of information on functional impairments in a cross-sectional study including patients with suspected but doubtful early IA according to GPs. We observed that, among patients with suspected IA according to GPs, those with actual IA according to the rheumatologist had higher HAQ-scores, and that the discriminative ability of a binary answer to a single question on difficulties with dressing was equal to that of the total HAQ. Results were validated in independent cohorts of a similar setting, namely in between primary and secondary care. Together the results imply that easily obtainable information on functional impairments may be of value in facilitating early detection of IA.

The HAQ was originally designed for patient-reported outcome assessment and not as a diagnostic tool.[14] However, it is the most widely used and validated instrument in the field of rheumatology for measuring functional impairments.[15,24] The HAQ was neither designed to use in parts (and assess questions individually) when used as outcome measure. Despite these limitations, the current data suggest that a simple question on difficulties with dressing, as deduced from the total HAQ, is of discriminative relevance. The simplicity of a single question and binary answers (no vs. any difficulties with dressing) may facilitate the use of assessing functional impairments for diagnostic purposes in busy daily practice and as part of future internet or mobile app based tools for early identification of IA.

The best discriminating question was one related to hand function. Interestingly, over 80% of patients reported to have symptoms of the hands (Table 1). Presumably, GPs are more often doubtful about IA in hand joints since these are relatively small joints and synovitis is often

For peer review only - http://bmjopen.bmj.com/site/about/guidelines.xhtml

subtle. Alternatively, GPs may be more inclined to assess hand joints compared to other joints. Exploratory subgroup analyses limited to patients who indicated to have hand symptoms yielded similar results (data not shown).

The current study was, to the best of our knowledge, the first to investigate the discriminative ability of functional impairments measured by the HAQ in the context of identifying early IA. Several other tools to promote detection of early IA have been reported, including self-administered questionnaires and internet-based tools for primary care providers.[25-28] However, none of these were validated tools that incorporated patient-reported functional impairments. The best studied initiative, the Early Inflammatory Arthritis Detection Tool reported by Bell MJ et al,[25] contained one question on functional impairments in general, namely whether important activities were affected by joint problems. Although this tool showed good overall discriminative ability, it was externally validated only in a tertiary care setting.[29] Its usability in cases of diagnostic uncertainty remains undetermined.

It is unknown how patient-reported functional impairments currently influence referral policy in patients with suspected IA in primary care. Information on functional limitations is generally lacking in local guidelines and referral criteria.[18,30-36] The Dutch GP guideline on IA, for example, recommends asking patients about the impact of their joint complaints on their daily lives, but does not specify the information that should be collected and in which way this should be incorporated into decision-making and referral policy.[18]

Although we attempted to seek for evidence on simple methods that are helpful to identify IA, the AUC reached by ‘difficulties with dressing’ was fairly limited. It is not surprising that a single question did not perform equally accurate as the reference, i.e. evaluation of swollen joints with joint palpation. However, particularly in the current absence of other evidence-

For peer review only - http://bmjopen.bmj.com/site/about/guidelines.xhtml

based strategies, this simple question that requires almost no extra effort, time or resources may be of value in improving early identification of IA.

The main strengths of the current study are related to the unique easy-access setting of the EA*R*Cs intermediary between primary and secondary care. Firstly, the EA*R*Cs could be visited weekly without waiting list. Secondly, GPs in our regions were encouraged to refer any patient in whom they suspected but doubted about the presence of IA, resulting in a selection of patients in whom there is a lower and more uncertain suspicion of IA than would be the case at one of the more extensively studied Early Arthritis Clinics. The latter are secondary care facilities intended for patients with clear presence or a very high suspicion of IA. Other strengths are the validation of our findings in two cohorts and critical evaluation of the added clinical relevance by adjusting for previously reported diagnostic variables.

A limitation of our study is that data were not collected in primary care practices. Given the relatively low incidence of IA in primary care, it must be noted that prospective research on this subject in general practices would be particularly challenging and resource-intensive. Still, validation in primary care is the next step to be taken. Although the sensitivity and specificity are expected to remain stable across differences in prevalence of IA, positive and negative predictive values are dependent on prior risks and may differ in primary care. Another limitation is that it is unknown how many patients were referred to but did not visit the EA*R*C. Although we assumed this number to be relatively low because of the accessible nature of the EA*R*Cs, it may have influenced the prevalence of IA in the study population. Nevertheless, the prevalence observed in this study suggests that GPs in our region already perform quite well in prioritising referrals, since their suspicion of IA was correct in almost half of referred patients. This is in line with previous findings from the EA*R*C.[16,21] Finally, as data were collected cross-sectionally and not longitudinally, analyses were not stratified for final diagnoses and patients who would develop RA were not studied separately.

For peer review only - http://bmjopen.bmj.com/site/about/guidelines.xhtml

On the other hand, patients with other types of IA (e.g. spondylo-, psoriatic and undifferentiated arthritis) will most likely benefit from early treatment and referral to a rheumatologist as well.[3-5]

In conclusion, we studied the discriminative ability of functional limitations measured by the HAQ and individual HAQ-questions in patients with suspected early IA. One question on difficulties with dressing by itself had a discriminative ability for actual presence of IA that equalled the full HAQ score and was validated in two cohorts. Although further research in primary care settings is necessary, the current data illustrate that a binary response to the question “Are you able to dress yourself, including shoelaces and buttons?” is helpful in the assessment of patients with suspected early IA and could be used in future internet or mobile app based tools aiming for early identification of IA.

For peer review only

DECLARATONS

Acknowledgements

We thank Xanthe M.E. Matthijssen for her statistical assistance.

Contributorship

BTvD, HWvS and AHMvHM designed the study.

BTvD, HWvS, EB and AHMvHM contributed to data collection.

All authors were involved in interpretation of data.

BTvD, EN and AHMvHM drafted and edited the manuscript.

All authors critically revised the manuscript.

Funding

This work was supported by the European Research Council (ERC) under the European Union's Horizon 2020 research and innovation programme (Starting grant, agreement No. 714312), and the Dutch Arthritis Society.

These organisations were not involved in in the design, execution or reporting of this study.

Competing interests

EB as an employee of the UMCG received speaker fees and consulting fees from Roche in 2017 and 2018 which were paid to the UMCG (outside the submitted work).

Otherwise non declared.

Data sharing statement

Data are available from BTvD (e-mail; B.T.van_Dijk@lumc.nl) upon reasonable request.

For peer review only

REFERENCES

1. Monti S, Montecucco C, Bugatti S, Caporali R. Rheumatoid arthritis treatment: the earlier the better to prevent joint damage. *RMD Open*. 2015;1(Suppl 1):e000057.

2. van der Linden MPM, le Cessie S, Raza K, van der Woude D, Knevel R, Huizinga TWJ, et al. Long-term impact of delay in assessment of patients with early arthritis. *Arthritis & Rheumatism*. 2010;62(12):3537-46.

3. Carron P, Varkas G, Cypers H, Van Praet L, Elewaut D, Van den Bosch F. Anti-TNF-induced remission in very early peripheral spondyloarthritis: the CRESPA study. 2017;76(8):1389-95.

4. Lopez-Olivo MA, Kakpovbia-Eshareturi V, des Bordes JK, Barbo A, Christensen R, Suarez-Almazor ME. Treating Early Undifferentiated Arthritis: A Systematic Review and Meta-Analysis of Direct and Indirect Trial Evidence. *Arthritis care & research*. 2018;70(9):1355-65.

5. McLaughlin M, Ostor A. Early treatment of psoriatic arthritis improves prognosis. *The Practitioner*. 2014;258(1777):21-4, 3.

6. Combe B, Landewe R, Daien CI, Hua C, Aletaha D, Alvaro-Gracia JM, et al. 2016 update of the EULAR recommendations for the management of early arthritis. *Annals of the rheumatic diseases*. 2017;76(6):948-59.

7. Meyfroidt S, Stevens J, De Lepeleire J, Westhovens R, De Cock D, Van der Elst K, et al. A general practice perspective on early rheumatoid arthritis management: A qualitative study from Flanders. *Eur J Gen Pract*. 2015;21(4):231-7.

8. NIVEL. Zorgregistraties eerste lijn: Incidenties en prevalenties [Internet]. 2017. https://www.nivel.nl/nl/zorgregistraties-eerste-lijn/incidenties-en-prevalenties (visited on 5 Feb 2019).

9. Jordan KP, Jöud A, Bergknut C, Croft P, Edwards JJ, Peat G, et al. International comparisons of the consultation prevalence of musculoskeletal conditions using population-based healthcare data from England and Sweden. *Ann Rheum Dis*. 2014;73(1):212-8.

10. Jordan KP, Kadam UT, Hayward R, Porcheret M, Young C, Croft P. Annual consultation prevalence of regional musculoskeletal problems in primary care: an observational study. *BMC Musculoskelet Disord*. 2010;11(1):144.

11. Raza K, Stack R, Kumar K, Filer A, Detert J, Bastian H, et al. Delays in assessment of patients with rheumatoid arthritis: variations across Europe. *Ann Rheum Dis*. 2011;70(10):1822-5.

12. Krebs P, Duncan DT. Health App Use Among US Mobile Phone Owners: A National Survey. *JMIR mHealth uHealth*. 2015;3(4):e101.

13. Subhi Y, Bube SH, Rolskov Bojsen S, Skou Thomsen AS, Konge L. Expert Involvement and Adherence to Medical Evidence in Medical Mobile Phone Apps: A Systematic Review. *JMIR mHealth uHealth*. 2015;3(3):e79.

14. Fries JF, Spitz P, Kraines RG, Holman HR. Measurement of patient outcome in arthritis. *Arthritis Rheum*. 1980;23(2):137-45.

15. Bruce B, Fries JF. The Stanford Health Assessment Questionnaire: dimensions and practical applications. *Health Qual Life Outcomes*. 2003;1:20.

For peer review only - http://bmjopen.bmj.com/site/about/guidelines.xhtml

16. van Nies JA, Brouwer E, van Gaalen FA, Allaart CF, Huizinga TW, Posthumus MD, et al. Improved early identification of arthritis: evaluating the efficacy of Early Arthritis Recognition Clinics. *Annals of the rheumatic diseases*. 2013;72(8):1295-301.

17. Newsum EC, de Waal MWM, van Steenbergen HW, Gussekloo J, van der Helm-van Mil AHM. How do general practitioners identify inflammatory arthritis? A cohort analysis of Dutch general practitioner electronic medical records. *Rheumatology (Oxford)*. 2016;55(5):848-53.

18. Nederlands Huisartsen Genootschap. NHG-Standaard Artritis (eerste herziening) [Internet]. 2017. https://www.nhg.org/standaarden/volledig/nhg-standaard-artritis (visited on 5 Feb 2019).

19. Ahlmen M, Svensson B, Albertsson K, Forslind K, Hafstrom I. Influence of gender on assessments of disease activity and function in early rheumatoid arthritis in relation to radiographic joint damage. *Ann Rheum Dis*. 2010;69(1):230-3.

20. Krishnan E, Sokka T, Hakkinen A, Hubert H, Hannonen P. Normative values for the Health Assessment Questionnaire disability index: benchmarking disability in the general population. *Arthritis Rheum*. 2004;50(3):953-60.

21. ten Brinck RM, van Dijk BT, van Steenbergen HW, le Cessie S, Numans ME, Hider SL, et al. Development and validation of a clinical rule for recognition of early inflammatory arthritis. *BMJ Open*. 2019;8(11):e023552.

22. White IR, Royston P, Wood AM. Multiple imputation using chained equations: Issues and guidance for practice. *Stat Med*. 2011;30(4):377-99.

23. Marshall A, Altman DG, Holder RL, Royston P. Combining estimates of interest in prognostic modelling studies after multiple imputation: current practice and guidelines. *BMC Med Res Methodol*. 2009;9:57.

24. Bruce B, Fries JF. The Stanford Health Assessment Questionnaire: a review of its history, issues, progress, and documentation. *J Rheumatol*. 2003;30(1):167-78.

25. Bell MJ, Tavares R, Guillemin F, Bykerk VP, Tugwell P, Wells GA. Development of a self-administered early inflammatory arthritis detection tool. *BMC Musculoskelet Disord*. 2010;11:50.

26. Khraishi M, Uphall E, Mong J. The self-administered rheumatoid arthritis (RA) screening questionnaire (RASQ) is a simple and effective tool to detect RA patients. *Ann Rheum Dis*. 2010;69 (suppl 3):374.

27. Maksymowych WP, Russell A, Homik J, Keeling S. E-triage Rheumatoid Arthritis Study in Early Arthritis (ERASE) [Internet]. 2007. http://www.arthritisdoctor.ca/erase-intro.php (visited on 28 March 2019).

28. Singh S, Kumar A, Panneerselvam K, Vennila JJ. Diagnosis of arthritis through fuzzy inference system. *J Med Syst*. 2012;36(3):1459-68.

29. Tavares R, Huang S, Bykerk VP, Bell MJ. A parallel group cohort to determine the measurement properties of an early inflammatory arthritis detection tool. *Rheumatology (Oxford)*. 2013;52(11):2077-85.

30. Almoallim H, Janoudi N, Attar SM, Garout M, Algohary S, Siddiqui MI, et al. Determining early referral criteria for patients with suspected inflammatory arthritis presenting to primary care physicians: a cross-sectional study. *Open Access Rheumatol*. 2017;9:81-90.

For peer review only - http://bmjopen.bmj.com/site/about/guidelines.xhtml

31. Croydon Health Services NHS Foundation Trust. Early arthritis services [Internet]. https://www.croydonhealthservices.nhs.uk/a-to-z-of-services/service/early-arthritis-service-151/ (visited on 19 March 2019).

32. Emery P, Breedveld FC, Dougados M, Kalden JR, Schiff MH, Smolen JS. Early referral recommendation for newly diagnosed rheumatoid arthritis: evidence based development of a clinical guide. *Ann Rheum Dis*. 2002;61(4):290-7.

33. Hampshire Hospitals NHS Foundation Trust. Early Arthritis Clinic referral form [Internet]. https://www.hampshirehospitals.nhs.uk/media/616367/updated_eac_form.pdf (visited on 19 March 2019).

34. Hülsemann J, Mattussek S, Siegmund-Schultze E, Zeidler H. [Early diagnosis of arthritis and spondyloarthritis within the framework of integrated health care in Lower Saxony]. *Z Rheumatol*. 2006;65(1):70-4.

35. Musgrove Park Hospital. Early Arthritis Referral Form [Internet]. https://www.tsft.nhs.uk/media/353049/early-arthritis-final.pdf (visited on 19 March 2019).

36. The National Institute for Health and Care Excellence (NICE). Rheumatoid arthritis in adults: management [Internet]. 2018. https://www.nice.org.uk/guidance/ng100 (visited on 19 March 2019).

For peer review only

For peer review only - http://bmjopen.bmj.com/site/about/guidelines.xhtml

TABLES AND FIGURES

Table 1. Characteristics of patients visiting the Early Arthritis *Recognition* Clinics

	**Derivation cohort (n = 997)**	**1st Validation cohort (n = 506)**	**2nd Validation cohort (n = 557)**
**Inclusion period, years**	2010–2014	2010–2014	2015–2018
**Male, n (%)**	290 (29)	177 (35)	183 (33)
**Age in years, mean ± SD**	51 ± 16	52 ± 16	54 ± 16
**Symptom duration in weeks, median (IQR)**	12 (4–62)	18 (5–69)	13 (4–57)
**Acute onset of symptoms, n (%)**	368 (38)	187 (38)	203 (38)
**Morning stiffness in minutes, median (IQR)**	10 (0–30)	10 (0–31)	10 (0–30)
**Number of painful joints, median (IQR)**	7 (3–15)	9 (4–18)	8 (2–17)
**Number of patient-reported swollen joints, median (IQR)**	2 (1–5)	3 (1–8)	2 (1–5)
**Difficulties with making a fist, n (%)**	242 (47)	130 (54)	291 (54)
**Arthralgia location, n (%)**			
≥1 hand or wrist joint	810 (81)	424 (84)	455 (82)
≥1 forefoot joint	254 (25)	142 (28)	152 (27)
≥1 hand, wrist or forefoot joint	869 (87)	454 (90)	486 (87)
Exclusively large joints*	22 (2)	18 (4)	9 (2)
**HAQ-score, mean ± SD**	0.77 ± 0.59	0.88 ± 0.67	–**
**IA present at joint examination, n (%)**	426 (43)	267 (53)	193 (35)

Legend

Original, unimputed data

* Shoulder, elbow, hip, knee or ankle

** Patients in the second validation cohort did not complete the full HAQ but only the question on difficulties with dressing.

Abbreviations: SD = standard deviation; IQR = interquartile range; IA = inflammatory arthritis.

For peer review only - http://bmjopen.bmj.com/site/about/guidelines.xhtml

Table 2. Association between presence of IA and the degree of difficulties with dressing in the derivation cohort (n = 997), with and without dichotomisation into binary scores

Difficulties with dressing		IA n (%)	No IA n (%)	OR (95%CI)	AUC (95%CI)
**Categorical***	0	169 (40)	310 (54)	1.00 (ref)	0.58 (0.55–0.62)
	1	188 (44)	210 (37)	1.64 (1.24–2.16)	
	2	62 (15)	45 (8)	2.49 (1.61–3.84)	
	3	7 (2)	6 (1)	2.20 (0.73–6.66)	
**Binary**	no (0)	169 (40)	310 (54)	1.00 (ref)	0.57 (0.54–0.61)
	yes (≥ 1)	257 (60)	261 (46)	1.80 (1.39–2.33)	

Legend

Results were pooled across 30 imputations; percentages and numbers were rounded.

* 0 = 'without any difficulty'; 1 = 'with some difficulty'; 2 = 'with much difficulty'; 3 = 'unable to do'

Abbreviations: IA = inflammatory arthritis; OR = odds ratio; CI = confidence interval; AUC = area under the receiver operating characteristics curve; ref = reference.

For peer review only

For peer review only - http://bmjopen.bmj.com/site/about/guidelines.xhtml

Table 3. Presence of difficulties with dressing associated with presence of IA independently from other clinical variables.

	OR (95%CI) univariable	OR (95%CI) age, gender adjusted	OR (95%CI) multivariable*
**Derivation cohort (n = 997)**	1.80 (1.39–2.33)	1.83 (1.41–2.39)	1.71 (1.27–2.32)
**1st Validation cohort (n = 506)**	2.00 (1.39–2.87)	1.89 (1.31–2.72)	1.64 (1.08–2.50)
**2nd Validation cohort (n = 557)**	2.14 (1.48–3.10)	1.95 (1.32–2.86)	1.87 (1.20–2.92)

Legend

Results were pooled across 30 imputations.

* Adjusted for: being male, age ≥60 years, symptom duration, acuteness of symptom onset, morning stiffness >60 minutes, number of painful joints, presence of patient-reported swollen joint(s) and difficulties with making a fist (see supplementary Table S2 for details).

Abbreviations: IA = inflammatory arthritis; OR = odds ratio; CI = confidence interval.

For peer review only

Figure 1. Test characteristics of the presence of difficulties with dressing (scores ≥1), with IA at physical examination as outcome

IMAGE UPLOADED SEPARATELY

Legend

Results were pooled across 30 imputations.

Error bars indicate the 95% confidence interval.

Abbreviations: IA = Inflammatory arthritis; Sens. = sensitivity; Spec. = specificity; PPV = positive predictive value; NPV = negative predictive value.

For peer review only

For peer review only - http://bmjopen.bmj.com/site/about/guidelines.xhtml

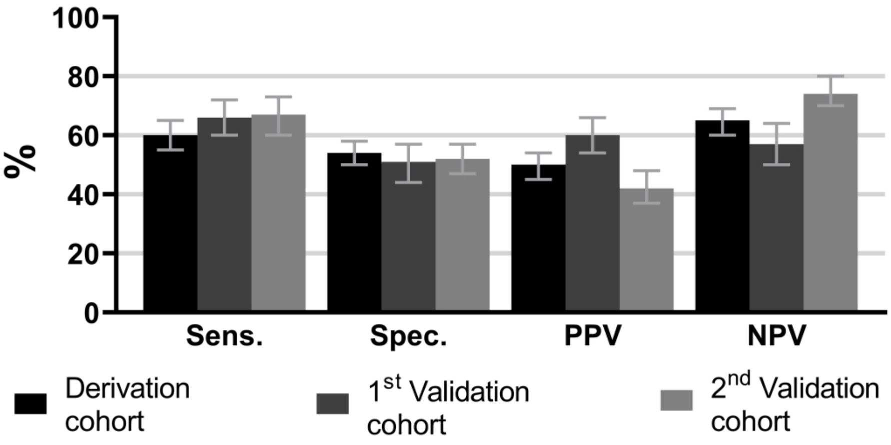

Test characteristics of the presence of difficulties with dressing (scores ≥1), with IA at physical examination as outcome

169x83mm (600 x 600 DPI)

Supplementary file

Table S1. Discriminative ability for the presence of IA of the total HAQ-score and its individual questions in the derivation cohort (n = 997)

Category	Item*	AUC (95%CI)	p
–	Total HAQ-score	0.55 (0.52–0.59)	0.006
**(1) Dressing & grooming**	(A) … Dress yourself, including shoelaces and buttons?	0.58 (0.55–0.62)	<0.001
	(B) … Wash your hair?	0.52 (0.48–0.56)	0.301
**(2) Arising**	(A) … Stand up from a straight chair?	0.55 (0.51–0.58)	0.016
	(B) … Get in and out of bed?	0.53 (0.49–0.57)	0.104
**(3) Eating**	(A) … Cut meat?	0.50 (0.46–0.54)	0.897
	(B) … Lift a full cup or glass to your mouth?	0.51 (0.47–0.55)	0.619
	(C) … Open a new milk carton?	0.48 (0.45–0.52)	0.372
**(4) Walking**	(A) … Walk outdoors on flat ground?	0.57 (0.53–0.60)	0.001
	(B) … Climb up five steps?	0.56 (0.52–0.59)	0.003
**(5) Hygiene**	(A) … Wash and dry your body?	0.54 (0.50–0.58)	0.041
	(B) … Get in and out of a tub bath?	0.53 (0.49–0.57)	0.138
	(C) … Get on and off the toilet?	0.56 (0.52–0.59)	0.003
**(6) Reach**	(A) … Reach and get down a 2½kg object (such as a bag of sugar) from above your head?	0.51 (0.47–0.55)	0.567
	(B) … Bend down to pick up clothing from the floor?	0.52 (0.48–0.56)	0.300
**(7) Grip**	(A) … Open car doors?	0.53 (0.49–0.57)	0.145
	(B) … Open previously opened jars?	0.49 (0.45–0.52)	0.464
	(C) … Turn a faucet on and off?	0.51 (0.47–0.55)	0.514
**(8) Activities**	(A) … Run errands and shop?	0.55 (0.51–0.59)	0.007
	(B) … Get in and out of a car?	0.54 (0.50-0.58)	0.037
	(C) … Do chores such as vacuuming or yard work?	0.55 (0.51–0.59)	0.011

Legend

* Names of categories are shown, as well as individual questions (at the EA*R*C, Dutch translations were used). Each question starts with ‘Are you able to …’. Patients indicated the degree of difficulties on each question with ‘without any difficulty’ (0), ‘with some difficulty’ (1), ‘with much difficulty’ (2) or ‘unable to do’ (3).

For peer review only

Abbreviations: IA = inflammatory arthritis; HAQ = Health Assessment Questionnaire Disability Index; AUC = area under the receiver operating characteristics curve; CI = confidence interval.

For peer review only

Table S2. Presence of difficulties with dressing associated with presence of IA independently from other clinical variables* (detailed overview of multivariable model)

		Derivation cohort (n = 997) OR (95%CI)	1st Validation cohort (n = 506) OR (95%CI)	2nd Validation cohort (n = 557) OR (95%CI)
**Male**		1.99 (1.47–2.70)	1.36 (0.91–2.03)	2.47 (1.66–3.68)
**Age ≥ 60 years**		1.82 (1.35–2.45)	1.40 (0.93–2.10)	1.90 (1.28–2.83)
**Symptom duration in weeks**	≥ 52	1.00 (ref)	1.00 (ref)	1.00 (ref)
	0–5.99	3.63 (2.45–5.39)	1.48 (0.86–2.53)	2.39 (1.38–4.17)
	6–51.99	2.16 (1.50–3.13)	1.24 (0.77–1.99)	1.92 (1.13–3.27)
**Acute onset of symptoms**		0.97 (0.72–1.32)	1.05 (0.69–1.58)	1.19 (0.77–1.83)
**Morning stiffness > 60 minutes**		1.34 (0.84–2.15)	1.59 (0.86–2.93)	0.84 (0.45–1.57)
**Number of painful joints**	0	1.00 (ref)	0.00**	1.00 (ref)
	1–3	4.45 (1.36–14.53)	1.54 (0.95–2.50)	2.70 (0.66–11.00)
	≥ 4	2.34 (0.73–7.53)	1.00 (ref)	1.60 (0.40–6.44)
**Patient-reported swollen joint(s) ≥ 1**		1.97 (1.27–3.04)	1.76 (0.95–3.25)	1.74 (1.07–2.81)
**Difficulties with making a fist**		1.31 (0.92–1.88)	1.54 (0.94–2.54)	1.57 (1.01–2.46)
**Difficulties with dressing**	yes (≥ 1)	1.71 (1.27–2.32)	1.64 (1.08–2.50)	1.87 (1.20–2.92)

Legend

Results were pooled across 30 imputations.

* These variables were selected based on a recent study from the Leiden EA*R*C that determined clinical variables that associated with the presence of IA.

** In the Groningen validation cohort none of the patients who indicated no painful joints on the questionnaire were positive for IA upon joint examination. Therefore, no meaningful CI could be calculated for this group and patients with ≥4 painful joints were assigned as reference group instead.

Abbreviations: IA = inflammatory arthritis; OR = odds ratio; CI = confidence interval, ref = reference.

For peer review only

Table S3. Characteristics of patients with and without available data on difficulties with dressing

	Derivation cohort			1st Validation cohort			2nd Validation cohort		
**Data on difficulties with dressing:**	**Available (n = 956)**	**Missing (n = 41)**	**p***	**Available (n = 495)**	**Missing (n = 11)**	**p***	**Available (n = 542)**	**Missing (n = 15)**	**p***
**Male, n (%)**	275 (29)	15 (37)	0.294	174 (35)	3 (27)	0.755	180 (33)	3 (20)	0.406
**Age in years, mean ± SD**	51 ± 16	57 ± 17	0.015	51 ± 16	57 ± 10	0.237	54 ± 16	58 ± 12	0.312
**Symptom duration in weeks, median (IQR)**	13 (4–62)	9 (3–72)	0.792	18 (5–69)	7 (5–64)	0.519	13 (4–59)	13 (5–24)	0.750
**Acute onset of symptoms, n (%)**	353 (38)	15 (41)	0.731	185 (38)	2 (20)	0.333	201 (37)	2 (29)	0.713
**Morning stiffness in minutes, median (IQR)**	10 (0–30)	4 (0–60)	0.983	10 (0–33)	10 (1–190)	0.776	10 (0–30)	0 (0–1)	0.012
**Number of painful joints, median (IQR)**	7 (3–14)	6 (2–25)	0.941	9 (4–18)	9 (3–20)	0.840	8 (3–17)	1 (1–9)	0.021
**Number of patient-reported swollen joints, median (IQR)**	2 (1–5)	2 (0–5)	0.503	3 (1–8)	5 (2–8)	0.698	2 (1–5)	1 (0–5)	0.317
**Difficulties with making a fist, n (%)**	239 (48)	3 (23)	0.095	128 (54)	2 (50)	1.000	288 (55)	3 (50)	1.000

Legend

Percentages are based on available questionnaire entries.

* Unpaired t-tests, Fisher's Exact tests and Mann-Whitney U tests were used as appropriate.

Abbreviations: SD = standard deviation; IQR = interquartile range.

For peer review only

Table S4. (sensitivity analyses limited to unimputed data) Association between presence of difficulties with dressing and presence of IA in the derivation and validation cohorts

	OR (95%CI) univariable	OR (95%CI) age, gender adjusted	OR (95%CI) multivariable*
**Derivation cohort**	1.83 (1.41–2.38)	1.87 (1.43–2.44)	1.59 (0.98–2.57)
**1st Validation cohort**	2.02 (1.41–2.91)	1.92 (1.33–2.77)	1.70 (0.83–3.84)
**2nd Validation cohort**	2.19 (1.51–3.17)	1.99 (1.36–2.93)	1.91 (1.16–3.16)

Legend

* Point estimates are similar to those obtained in the main analyses, but have broader confidence intervals since only complete cases could be analysed. Covariates: being male, age ≥60 years, symptom duration, acuteness of symptom onset, morning stiffness >60 minutes, number of painful joints, presence of patient-reported swollen joint(s) and difficulties with making a fist.

Abbreviations: IA = inflammatory arthritis; OR = odds ratio; CI = confidence interval.

For peer review only

STROBE 2007 (v4) Statement—Checklist of items that should be included in reports of *cross-sectional studies*

Section/Topic	Item #	Recommendation	Reported on page #
**Title and abstract**	1	(*a*) Indicate the study's design with a commonly used term in the title or the abstract	1, 2
		(*b*) Provide in the abstract an informative and balanced summary of what was done and what was found	2
**Introduction**			
Background/rationale	2	Explain the scientific background and rationale for the investigation being reported	5, 6
Objectives	3	State specific objectives, including any prespecified hypotheses	6
**Methods**			
Study design	4	Present key elements of study design early in the paper	6–8
Setting	5	Describe the setting, locations, and relevant dates, including periods of recruitment, exposure, follow-up, and data collection	6–8
Participants	6	(*a*) Give the eligibility criteria, and the sources and methods of selection of participants	6, 7
Variables	7	Clearly define all outcomes, exposures, predictors, potential confounders, and effect modifiers. Give diagnostic criteria, if applicable	8, 9
Data sources/ measurement	8*	For each variable of interest, give sources of data and details of methods of assessment (measurement). Describe comparability of assessment methods if there is more than one group	7
Bias	9	Describe any efforts to address potential sources of bias	9, 10
Study size	10	Explain how the study size was arrived at	7
Quantitative variables	11	Explain how quantitative variables were handled in the analyses. If applicable, describe which groupings were chosen and why	8, 9
Statistical methods	12	(*a*) Describe all statistical methods, including those used to control for confounding	9, 10
		(*b*) Describe any methods used to examine subgroups and interactions	*NA*
		(*c*) Explain how missing data were addressed	9, 10
		(*d*) If applicable, describe analytical methods taking account of sampling strategy	*NA*
		(*e*) Describe any sensitivity analyses	10
**Results**			

For peer review only

Participants	13*	(a) Report numbers of individuals at each stage of study—eg numbers potentially eligible, examined for eligibility, confirmed eligible, included in the study, completing follow-up, and analysed	21
		(b) Give reasons for non-participation at each stage	7
		(c) Consider use of a flow diagram	*NA*
Descriptive data	14*	(a) Give characteristics of study participants (eg demographic, clinical, social) and information on exposures and potential confounders	21
		(b) Indicate number of participants with missing data for each variable of interest	9
Outcome data	15*	Report numbers of outcome events or summary measures	10–12, 21–23
Main results	16	(*a*) Give unadjusted estimates and, if applicable, confounder-adjusted estimates and their precision (eg, 95% confidence interval). Make clear which confounders were adjusted for and why they were included	23
		(*b*) Report category boundaries when continuous variables were categorized	22
		(*c*) If relevant, consider translating estimates of relative risk into absolute risk for a meaningful time period	*NA*
Other analyses	17	Report other analyses done—eg analyses of subgroups and interactions, and sensitivity analyses	12
**Discussion**			
Key results	18	Summarise key results with reference to study objectives	13
Limitations	19	Discuss limitations of the study, taking into account sources of potential bias or imprecision. Discuss both direction and magnitude of any potential bias	15
Interpretation	20	Give a cautious overall interpretation of results considering objectives, limitations, multiplicity of analyses, results from similar studies, and other relevant evidence	16
Generalisability	21	Discuss the generalisability (external validity) of the study results	15, 16
**Other information**			
Funding	22	Give the source of funding and the role of the funders for the present study and, if applicable, for the original study on which the present article is based	17

*Give information separately for cases and controls in case-control studies and, if applicable, for exposed and unexposed groups in cohort and cross-sectional studies.

Note: An Explanation and Elaboration article discusses each checklist item and gives methodological background and published examples of transparent reporting. The STROBE checklist is best used in conjunction with this article (freely available on the Web sites of PLoS Medicine at http://www.plosmedicine.org/, Annals of Internal Medicine at http://www.annals.org/, and Epidemiology at http://www.epidem.com/). Information on the STROBE Initiative is available at www.strobe-statement.org.

For peer review only

BMJ Open

The value of inquiring about functional impairments for early identification of inflammatory arthritis: a large cross-sectional derivation and validation study

Journal:	*BMJ Open*
Manuscript ID	bmjopen-2020-040148.R1
Article Type:	Original research
Date Submitted by the Author:	22-Jul-2020
Complete List of Authors:	van Dijk, Bastiaan; Leiden University Medical Center,<br>van Steenbergen, Hanna; Leiden University Medical Center, Rheumatology<br>Niemantsverdriet, Ellis; Leiden University Medical Center, Rheumatology<br>Brouwer, Elisabeth; University Medical Centre Groningen, Rheumatology and Clinical Immunology<br>van der Helm-van Mil, Annette; Leiden University Medical Center, Rheumatology; Erasmus Medical Center, Rheumatology
**Primary Subject Heading**:	Rheumatology
Secondary Subject Heading:	General practice / Family practice
Keywords:	RHEUMATOLOGY, PRIMARY CARE, Rheumatology < INTERNAL MEDICINE

SCHOLARONE™
Manuscripts

I, the Submitting Author has the right to grant and does grant on behalf of all authors of the Work (as defined in the below author licence), an exclusive licence and/or a non-exclusive licence for contributions from authors who are: i) UK Crown employees; ii) where BMJ has agreed a CC-BY licence shall apply, and/or iii) in accordance with the terms applicable for US Federal Government officers or employees acting as part of their official duties; on a worldwide, perpetual, irrevocable, royalty-free basis to BMJ Publishing Group Ltd ("BMJ") its licensees and where the relevant Journal is co-owned by BMJ to the co-owners of the Journal, to publish the Work in this journal and any other BMJ products and to exploit all rights, as set out in our licence.

The Submitting Author accepts and understands that any supply made under these terms is made by BMJ to the Submitting Author unless you are acting as an employee on behalf of your employer or a postgraduate student of an affiliated institution which is paying any applicable article publishing charge ("APC") for Open Access articles. Where the Submitting Author wishes to make the Work available on an Open Access basis (and intends to pay the relevant APC), the terms of reuse of such Open Access shall be governed by a Creative Commons licence – details of these licences and which Creative Commons licence will apply to this Work are set out in our licence referred to above.

Other than as permitted in any relevant BMJ Author's Self Archiving Policies, I confirm this Work has not been accepted for publication elsewhere, is not being considered for publication elsewhere and does not duplicate material already published. I confirm all authors consent to publication of this Work and authorise the granting of this licence.

For peer review only

Original research

The value of inquiring about functional impairments for early identification of inflammatory arthritis: a large cross-sectional derivation and validation study

Bastiaan T. van Dijk[1], Hanna W. van Steenbergen[1], Ellis Niemantsverdriet[1], Elisabeth Brouwer[2], Annette H.M. van der Helm-van Mil[1,3]

[1] Department of Rheumatology, Leiden University Medical Centre, Leiden, the Netherlands

[2] Department of Rheumatology and Clinical Immunology, University Medical Centre Groningen, Groningen, the Netherlands

[3] Department of Rheumatology, Erasmus Medical Centre, Rotterdam, the Netherlands

Corresponding author

B.T. van Dijk, M.D., Department of Rheumatology, Leiden University Medical Centre

P.O. Box 9600, 2300 RC Leiden, the Netherlands

Tel.: +31 71 5265655; Email: B.T.van_Dijk@lumc.nl

ORCID ID: 0000-0002-5161-6791

Word count (main text): 3260

For peer review only - http://bmjopen.bmj.com/site/about/guidelines.xhtml

ABSTRACT

Objectives. Healthcare professionals other than rheumatologists experience difficulties in detecting early inflammatory arthritis (IA) by joint examination. Self-reported symptoms are increasingly considered as helpful and could be incorporated in online tools to assist healthcare professionals, but firstly their discriminative ability must be assessed. As part of this effort, we evaluated whether inquiring about functional impairments could aid early IA-identification.

Design. Cross-sectional derivation and validation study.

Setting. Data from two Early Arthritis *Recognition* Clinics (EA*R*C) in the Netherlands were studied, which are easy-access outpatient rheumatology clinics intermediary between primary and secondary care for patients in whom GPs suspect but are unsure about IA-presence.

Participants. Between 2010-2014, 997 patients consecutively visited the Leiden-EA*R*C (derivation cohort). Patients consecutively visiting the Groningen-EA*R*C (2010-2014, n=506) and Leiden-EA*R*C (2015-2018, n=557) served as validation cohorts.

Primary and secondary outcome measures. Physical functioning was assessed with the Health Assessment Questionnaire Disability-Index (HAQ); IA-presence by physical joint examination by rheumatologists. HAQ-questions were studied individually regarding discriminative ability for IA-presence. For the best discriminating question, odds ratios (ORs) and positive predictive values (PPVs) for IA-presence were determined.

Results. IA was ascertained in 43% (derivation cohort), 53% and 35% (validation cohorts). In the derivation cohort, IA-presence associated with higher mean HAQ-scores (0.84 versus 0.73, p=0.003). One question on difficulties with dressing equalled discriminative ability of the total HAQ-score. ‘Difficulties with dressing’ yielded ORs for IA-presence of 1.8(95%CI

For peer review only - http://bmjopen.bmj.com/site/about/guidelines.xhtml

1.4-2.4) in the derivation cohort; 2.0(1.4-2.9) and 2.1(1.5-3.1) in the validation cohorts. After adjustments for clinical characteristics these were 1.7(1.3-2.3), 1.6(1.1-2.5) and 1.9(1.2-2.9). PPVs (probabilities of IA for positive answers) ranged 42-60% and NPVs (probabilities of no IA for negative answers) ranged 57-74%.

Conclusions. Patient-reported difficulties with dressing in patients with suspected IA associated with actual IA-presence. Although further validation is required, e.g. in primary care, this simple question could be of help in future early IA-detection tools for healthcare professionals with limited experience in joint examination.

For peer review only

For peer review only - http://bmjopen.bmj.com/site/about/guidelines.xhtml

STRENGHTS AND LIMITATIONS OF THIS STUDY

- We evaluated the value of questions about functional impairments for early recognition of inflammatory arthritis (IA), for which new and simple tools are urgently needed – especially in primary care.
- This was studied in a setting intermediary between primary and secondary care that services patients whose general practitioners (GPs) were doubtful about the presence of IA; the population for whom such new tools are most relevant.
- One simple question about difficulties with dressing was associated with presence of IA, which equalled discriminative ability of the total HAQ but is more suitable for busy daily practice.
- This association was tested for independence from known predictors and validated in two independent cohorts.
- However, further validation in primary care is needed, since data were not yet collected in actual primary care practices.

For peer review only

INTRODUCTION

In rheumatoid arthritis (RA), early initiation of treatment with disease-modifying antirheumatic drugs (DMARDs) is associated with improved outcomes.[1,2] Also for other types of inflammatory arthritis (IA) the importance of early treatment is increasingly recognised.[3-5] To achieve early treatment initiation however, it is necessary for at-risk patients to be referred to a rheumatologist in a timely fashion.[6]

Consequently, general practitioners (GPs) play a crucial role in early identification of IA, but they have indicated considerable barriers regarding timely and adequate referral of patients with musculoskeletal complaints, including a lack of self-confidence in detecting synovitis.[7] GPs generally have limited experience with the detection of IA by joint palpation compared to rheumatologists, since their patient population is characterised by a relatively low incidence of RA and IA (0.7/1000 patient years in the Netherlands, which amounts to 1–2 new patients on average per GP per year) compared to a much higher incidence of other musculoskeletal complaints.[8-10] These circumstances may add to referral delay at the level of the initial healthcare provider (usually the GP), which was found to be an important contributor to overall delay until treatment initiation in many European countries.[11]

In this light, the search for novel strategies to improve early recognition and referral of patients at risk for IA and RA remains of great importance. This was emphasised in the latest update of the European League against Rheumatism (EULAR) research agenda for early arthritis by including 'the identification of tools that could help GPs diagnose early arthritis and prioritise referral.'[6] Given the increasing usage of healthcare-related mobile applications (apps)[12], new tools are preferred to be simple and time-efficient, providing suitability for possible future digital initiatives for early IA-screening. In addition to ease-of-

For peer review only - http://bmjopen.bmj.com/site/about/guidelines.xhtml

use, such tools should be based on valid and reliable data but currently most medical mobile apps are not properly evidence-based, highlighting the need for thorough research.[13]

Patients with IA generally experience limitations in physical functioning. One could hypothesise that these limitations may differ in sort and severity compared to those related to other, less urgent causes of joint symptoms. Functional impairments in patients with rheumatic diseases are generally assessed using the Health Assessment Questionnaire Disability-Index (HAQ),[14] but use of this patient-reported outcome instrument is mostly limited to research in established rheumatic disease instead of earlier stages. Nonetheless, it is generally considered a self-explanatory and relatively efficient questionnaire (it can be completed within five minutes).[15] In order to search for tools that might facilitate early identification of IA we performed a cross-sectional study on the association between functional impairments (measured by the HAQ) and presence of IA at joint examination (identified by rheumatologists). Aiming for simple and time-efficient use in daily practice, we also studied the HAQ-questions separately and evaluated whether a single question could facilitate early identification of IA. Although information on functional impairments alone is not expected to have sufficient accuracy, it may be used as part of a more comprehensive tool that could be incorporated in a screening app for early IA. Findings were verified in two validation cohorts.

PATIENTS AND METHODS

Patients

Aiming to decrease referral delay in early IA, Early Arthritis *Recognition* Clinics (EA*R*C) were initiated in 2010 at the Leiden and Groningen University Medical Centres (LUMC and UMCG) in the Netherlands. The EA*R*C design has been described previously.[16] In short,

For peer review only - http://bmjopen.bmj.com/site/about/guidelines.xhtml

EA*R*Cs are clinics for patients in whom their GP suspects but is unsure about the presence of IA. These easy-access clinics are characterised by their unique intermediate setting, namely in between primary and secondary care. The EA*R*Cs have no waiting lists, can be visited by referred patients once to twice a week without an appointment and have markedly reduced referral delay.[16] GPs in the region are instructed to quickly refer any patient for whom they are unsure about the presence of IA, instead of applying a strategy of 'watchful waiting' or performing additional diagnostic tests. Notably, auto-antibodies or other additional investigations are generally not performed by GPs in our region[17], which is in accordance with the Dutch national guidelines.[18]

At the EA*R*C, patients completed a brief clinical questionnaire on their symptoms, followed by a short consultation and full 66-joint examination by an experienced rheumatologist. If IA was detected at physical examination (by palpable joint swelling), patients were analysed at the regular rheumatology outpatient clinic for further diagnosis. Otherwise, they were referred back to their GP. Laboratory tests and imaging were not performed at the EA*R*C.

The derivation cohort comprised 997 patients who consecutively visited the Leiden-EA*R*C between September 2010 and April 2014. The first validation cohort consisted of 506 patients who visited the Groningen-EA*R*C from September 2010 to January 2014. The second validation cohort consisted of 557 patients who visited the Leiden-EA*R*C from November 2015 to December 2018. Patients who visited the Leiden-EA*R*C from April 2014 to November 2015 did not fill out questions on physical functioning and were not included in this study.

For peer review only - http://bmjopen.bmj.com/site/about/guidelines.xhtml

Patient involvement

Patient research partners agreed with the pathway of care at EA*R*C and provided feedback on the questionnaire, which was expanded in 2012 with two questions.

HAQ

Physical functioning was assessed during the same EA*R*C-visit using the HAQ, a questionnaire containing 20 questions on impairments across eight functional categories.[14,15] Each question was scored by patients on a 4-point scale ranging 0–3, representing the degree of difficulties experienced when performing the corresponding activity, with 0 indicating no difficulties and 3 indicating full disability. The total HAQ score is calculated as the average across eight categories and ranges 0–3. Patients in the derivation cohort and patients in the first validation cohort filled out the full HAQ. Based on initial results from the derivation cohort, patients in the second validation cohort were asked to complete only question 1A on difficulties with dressing.

Outcome

The outcome used in all statistical analyses was presence of IA, determined by the rheumatologist at physical examination. Final classifying diagnoses were made during subsequent visit(s) at the regular rheumatology outpatient clinic and were beyond the scope of this study. The rheumatologists who determined IA at joint examination were not formally blinded to the HAQ-data; at request however the participating rheumatologists answered not to consider HAQ-data in their evaluation of the presence of IA (personal communication).

For peer review only - http://bmjopen.bmj.com/site/about/guidelines.xhtml

Statistical analyses

In the derivation cohort, total HAQ-scores were compared between patients with and without IA at joint examination. Areas under the curve (AUCs) were determined for the total HAQ-score as well as its questions individually, in order to select the best discriminating question for further investigation (based on highest AUC). The use of aids and devices was taken into account for calculation of the total HAQ-score as prescribed, but not for scores on individual questions.[14,15] For the best discriminating question, odds ratios (ORs) and AUCs were determined with both the original, categorical responses and with binary responses (no vs. any degree of difficulties; i.e. scores ≥1), since a binary tool would be even more suited for easy use in daily practice. ORs were adjusted for age and gender.[19-21] Test characteristics and probabilities of IA were calculated. Analyses were repeated in the two validation cohorts.

A previous study from the Leiden-EA*R*C derived an algorithm for IA-identification, consisting of clinical variables that were independently associated with the presence of IA (being male, age ≥60 years, symptom duration, acuteness of symptom onset, morning stiffness >60 minutes, number of painful joints, presence of patient-reported swollen joint(s) and difficulties with making a fist).[21] These variables were included in multivariable logistic regression analyses, to study if self-reported functional disabilities were independently associated with IA. In addition, change in diagnostic accuracy when adding functional disability to the algorithm was assessed in the derivation cohort by comparing AUCs.

The HAQ-question of interest was missing in 4%, 2% and 3% of the cohorts respectively. Characteristics of patients with available and missing data on this HAQ-question were compared. Missing values were imputed using multiple imputation by chained equations (MICE) with predictive mean matching (PMM).[22] Rubin's rules were applied to pool point estimates and confidence intervals across 30 imputations.[23] Imputed data were used for the

For peer review only - http://bmjopen.bmj.com/site/about/guidelines.xhtml

main analyses. In sensitivity analyses, results were compared to those obtained from the unimputed data.

IBM SPSS (version 25) and R (version 3.5.2) were used. P-values <0.05 were considered statistically significant.

RESULTS

Patients

Patient characteristics for all three cohorts are presented in Table 1. Over 80% in each cohort presented with hand and/or wrist arthralgia. IA was diagnosed in 43%, 53% and 35% of patients, respectively. Patients in the first validation cohort had a slightly longer symptom duration (median 18 weeks) than patients in the derivation and second validation cohorts (median 12 and 13 weeks, respectively). All other characteristics were similar.

Discriminative ability of a single question equalled the total HAQ score

Patients with IA at physical examination had a higher mean total HAQ score than patients without IA in the derivation cohort (0.84 (95%CI 0.77–0.89) compared to 0.73 (0.67–0.77), p=0.003), indicating a higher degree of functional disability. A gradual increase of the probability of IA at joint examination was observed as total HAQ-scores rose (Figure 1).

To search for a method that is more simple and time-efficient for usage in daily practice than the 20-question total HAQ, its questions were also studied individually (supplementary Table S1). Of these questions, scores on 'difficulties with dressing' (question 1A) yielded the highest AUC, which equalled the AUC of the total HAQ score: 0.58 (0.55–0.62) vs. 0.55 (0.52–0.59), respectively. This indicates that, in addition to greater ease of use, the

For peer review only - http://bmjopen.bmj.com/site/about/guidelines.xhtml

discriminative ability of just one question was equal to the total HAQ. For these reasons, this question ("Are you able to dress yourself, including shoelaces and buttons?") was considered the best discriminating question and was studied further instead of the total HAQ-score. The percentage of patients with different categories of impairment among the patients with and without IA are presented in Table 2. The probabilities of IA-presence for the different categories of impairments with dressing are indicated in Figure 1B; the probability increased with more impairment.

Since a binary question is even easier to implement in practice, we assessed the discriminative ability of dichotomised scores on difficulties with dressing. Loss of overall discriminative ability, measured using the AUC, induced by dichotomisation into a binary score was minor (AUC of 0.57 (0.54–0.61) vs. 0.58 (0.55–0.62)). Observed probabilities of IA-presence with this dichotomised score are presented in Figure 1C. For the benefit of ease of use this minor difference was accepted and dichotomised scores were used in subsequent analyses.

Difficulties with dressing were independently associated with presence of IA in derivation cohort

ORs for the IA-presence of a confirmative answer on the question about difficulties with dressing are presented in Table 3. In the derivation cohort, the presence of difficulties with dressing (scores ≥1) was associated with presence of IA with an age and gender adjusted OR of 1.83 (1.41–2.39). After further adjustment for the aforementioned clinical variables that were previously identified as associated with IA (being male, age ≥60 years, symptom duration, acuteness of symptom onset, morning stiffness >60 minutes, number of painful joints, presence of patient-reported swollen joint(s) and difficulties with making a fist), difficulties with dressing remained independently associated with the presence of IA, with an

For peer review only - http://bmjopen.bmj.com/site/about/guidelines.xhtml

OR of 1.71 (1.27–2.32). ORs for the other clinical variables are provided in the supplementary file (supplementary Table S2). The AUC of the model with only the aforementioned clinical variables was 0.72 (0.69–0.76), while the model where difficulties with dressing was added had an AUC of 0.73 (0.70–0.77). Since the presence of difficulties with dressing was independently associated with IA it was subsequently studied in the validation cohorts.

Difficulties with dressing were independently associated with presence of IA in both validation cohorts

In the first and second validation cohorts, age and gender adjusted ORs were 1.89 (1.31–2.72) and 1.95 (1.32–2.86), respectively. After further adjustment for the aforementioned clinical variables that were previously identified as associated with IA, ORs were 1.64 (1.08–2.50) and 1.87 (1.20–2.92), respectively. Thus, the independent association of difficulties with dressing with presence of IA was conformed in both validation cohorts.

Predictive values and test characteristics

Lastly, we studied test characteristics and predictive values of positive answers on just the single question about difficulties with dressing (Figure 2). The negative predictive values (NPVs) in the different cohorts ranged between 57–74%, indicating that 57–74% of patients without difficulties with dressing had no IA at physical examination. The positive predictive values (PPVs) ranged between 42–60%, meaning that 42–60% of patients who reported to have difficulties with dressing were indeed identified as having IA.

For peer review only - http://bmjopen.bmj.com/site/about/guidelines.xhtml

Sensitivity analyses on unimputed data

No clinically relevant differences in characteristics of patients with and without information on ‘difficulties with dressing’ were found (supplementary Table S3). Sub-analyses limited to the unimputed data (supplementary Table S4) yielded similar results as in the main analyses.

DISCUSSION

Healthcare professionals who are not rheumatologists generally have limited experience in joint examination, which hampers them in early identification of IA. For GPs this contributes to referral delay and necessitates the development of feasible tools that could support early identification of IA. We investigated the value of information on functional impairments in a cross-sectional study including patients with suspected but doubtful early IA according to GPs. We observed that, among patients with suspected IA according to GPs, those with actual IA according to the rheumatologist had higher HAQ-scores, and that the discriminative ability of a binary answer to a single question on difficulties with dressing was equal to that of the total HAQ. Results were validated in independent cohorts of a similar setting, namely in between primary and secondary care. Together the results imply that easily obtainable information on functional impairments may be of value in facilitating early detection of IA.

The HAQ was originally designed for patient-reported outcome assessment and not as a diagnostic tool.[14] However, it is the most widely used and validated instrument in the field of rheumatology for measuring functional impairments.[15,24] The HAQ was neither designed to use in parts (and assess questions individually) when used as outcome measure. Despite these limitations, the current data suggest that a simple question on difficulties with dressing, as deduced from the total HAQ, is of discriminative relevance. The simplicity of a single question and binary answers (no vs. any difficulties with dressing) may facilitate the

For peer review only - http://bmjopen.bmj.com/site/about/guidelines.xhtml

use of assessing functional impairments for diagnostic purposes in busy daily practice and as part of future internet or mobile app based tools for early identification of IA.

The best discriminating question was one related to hand function. Interestingly, over 80% of patients reported to have symptoms of the hands (Table 1). Presumably, GPs are more often doubtful about IA in hand joints since these are relatively small joints and synovitis is often subtle. Alternatively, GPs may be more inclined to assess hand joints compared to other joints. Exploratory subgroup analyses limited to patients who indicated to have hand symptoms yielded similar results (data not shown).

The current study was, to the best of our knowledge, the first to investigate the discriminative ability of functional impairments measured by the HAQ in the context of identifying early IA. Several other tools to promote detection of early IA have been reported, including self-administered questionnaires and internet-based tools for primary care providers.[25-28] However, none of these were validated tools that incorporated patient-reported functional impairments. The best studied initiative, the Early Inflammatory Arthritis Detection Tool reported by Bell MJ et al,[25] contained one question on functional impairments in general, namely whether important activities were affected by joint problems. Although this tool showed good overall discriminative ability, it was externally validated only in a tertiary care setting.[29] Its usability in cases of diagnostic uncertainty remains undetermined.

It is unknown how patient-reported functional impairments currently influence referral policy in patients with suspected IA in primary care. Information on functional limitations is generally lacking in local guidelines and referral criteria.[18,30-36] The Dutch GP guideline on IA, for example, recommends asking patients about the impact of their joint complaints on their daily lives, but does not specify the information that should be collected and in which way this should be incorporated into decision-making and referral policy.[18]

For peer review only - http://bmjopen.bmj.com/site/about/guidelines.xhtml

Although we attempted to seek for evidence on simple methods that are helpful to identify IA, the AUC reached by 'difficulties with dressing' was fairly limited. It is not surprising that a single question did not perform equally accurate as the reference, i.e. evaluation of swollen joints with joint palpation. However, particularly in the current absence of other evidence-based strategies, this simple question that requires almost no extra effort, time or resources may be of value in improving early identification of IA.

The main strengths of the current study are related to the unique easy-access setting of the EA*R*Cs intermediary between primary and secondary care. Firstly, the EA*R*Cs could be visited weekly without waiting list. Secondly, GPs in our regions were encouraged to refer any patient in whom they suspected but doubted about the presence of IA, resulting in a selection of patients in whom there is a lower and more uncertain suspicion of IA than would be the case at one of the more extensively studied Early Arthritis Clinics. The latter are secondary care facilities intended for patients with clear presence or a very high suspicion of IA. Other strengths are the validation of our findings in two cohorts and critical evaluation of the added clinical relevance by adjusting for previously reported diagnostic variables.

A limitation of our study is that data were not collected in primary care practices. Given the relatively low incidence of IA in primary care, it must be noted that prospective research on this subject in general practices would be particularly challenging and resource-intensive. Still, validation in primary care is the next step to be taken. Although the sensitivity and specificity are expected to remain stable across differences in prevalence of IA, positive and negative predictive values are dependent on prior risks and may differ in primary care. Another limitation is that it is unknown how many patients were referred to but did not visit the EA*R*C. Although we assumed this number to be relatively low because of the accessible nature of the EA*R*Cs, it may have influenced the prevalence of IA in the study population. Nevertheless, the prevalence observed in this study suggests that GPs in our region already

For peer review only - http://bmjopen.bmj.com/site/about/guidelines.xhtml

perform quite well in prioritising referrals, since their suspicion of IA was correct in almost half of referred patients. This is in line with previous findings from the EA*R*C.[16,21] Finally, as data were collected cross-sectionally and not longitudinally, analyses were not stratified for final diagnoses and patients who would develop RA were not studied separately. On the other hand, patients with other types of IA (e.g. spondylo-, psoriatic and undifferentiated arthritis) will most likely benefit from early treatment and referral to a rheumatologist as well.[3-5]

In conclusion, we studied the discriminative ability of functional limitations measured by the HAQ and individual HAQ-questions in patients with suspected early IA. One question on difficulties with dressing by itself had a discriminative ability for actual presence of IA that equalled the full HAQ score and was validated in two cohorts. Although further research in primary care settings is necessary, the current data illustrate that a binary response to the question “Are you able to dress yourself, including shoelaces and buttons?” is helpful in the assessment of patients with suspected early IA and could be used in future internet or mobile app based tools aiming for early identification of IA.

For peer review only

DECLARATONS

Acknowledgements

We thank Xanthe M.E. Matthijssen for her statistical assistance.

Contributorship

BTvD, HWvS and AHMvHM designed the study.

BTvD, HWvS, EB and AHMvHM contributed to data collection.

All authors were involved in interpretation of data.

BTvD, EN and AHMvHM drafted and edited the manuscript.

All authors critically revised the manuscript.

Funding

This work was supported by the European Research Council (ERC) under the European Union's Horizon 2020 research and innovation programme (Starting grant, agreement No. 714312), and the Dutch Arthritis Society.

These organisations were not involved in in the design, execution or reporting of this study.

Competing interests

EB as an employee of the UMCG received speaker fees and consulting fees from Roche in 2017 and 2018 which were paid to the UMCG (outside the submitted work).

Otherwise non declared.

Data sharing statement

Data are available from BTvD (e-mail; B.T.van_Dijk@lumc.nl) upon reasonable request.

For peer review only - http://bmjopen.bmj.com/site/about/guidelines.xhtml

REFERENCES

1. Monti S, Montecucco C, Bugatti S, Caporali R. Rheumatoid arthritis treatment: the earlier the better to prevent joint damage. *RMD Open*. 2015;1(Suppl 1):e000057.

2. van der Linden MPM, le Cessie S, Raza K, van der Woude D, Knevel R, Huizinga TWJ, et al. Long-term impact of delay in assessment of patients with early arthritis. *Arthritis & Rheumatism*. 2010;62(12):3537-46.

3. Carron P, Varkas G, Cypers H, Van Praet L, Elewaut D, Van den Bosch F. Anti-TNF-induced remission in very early peripheral spondyloarthritis: the CRESPA study. 2017;76(8):1389-95.

4. Lopez-Olivo MA, Kakpovbia-Eshareturi V, des Bordes JK, Barbo A, Christensen R, Suarez-Almazor ME. Treating Early Undifferentiated Arthritis: A Systematic Review and Meta-Analysis of Direct and Indirect Trial Evidence. *Arthritis care & research*. 2018;70(9):1355-65.

5. McLaughlin M, Ostor A. Early treatment of psoriatic arthritis improves prognosis. *The Practitioner*. 2014;258(1777):21-4, 3.

6. Combe B, Landewe R, Daien CI, Hua C, Aletaha D, Alvaro-Gracia JM, et al. 2016 update of the EULAR recommendations for the management of early arthritis. *Annals of the rheumatic diseases*. 2017;76(6):948-59.

7. Meyfroidt S, Stevens J, De Lepeleire J, Westhovens R, De Cock D, Van der Elst K, et al. A general practice perspective on early rheumatoid arthritis management: A qualitative study from Flanders. *Eur J Gen Pract*. 2015;21(4):231-7.

8. NIVEL. Zorgregistraties eerste lijn: Incidenties en prevalenties [Internet]. 2017. https://www.nivel.nl/nl/zorgregistraties-eerste-lijn/incidenties-en-prevalenties (visited on 5 Feb 2019).

9. Jordan KP, Jöud A, Bergknut C, Croft P, Edwards JJ, Peat G, et al. International comparisons of the consultation prevalence of musculoskeletal conditions using population-based healthcare data from England and Sweden. *Ann Rheum Dis*. 2014;73(1):212-8.

10. Jordan KP, Kadam UT, Hayward R, Porcheret M, Young C, Croft P. Annual consultation prevalence of regional musculoskeletal problems in primary care: an observational study. *BMC Musculoskelet Disord*. 2010;11(1):144.

11. Raza K, Stack R, Kumar K, Filer A, Detert J, Bastian H, et al. Delays in assessment of patients with rheumatoid arthritis: variations across Europe. *Ann Rheum Dis*. 2011;70(10):1822-5.

12. Krebs P, Duncan DT. Health App Use Among US Mobile Phone Owners: A National Survey. *JMIR mHealth uHealth*. 2015;3(4):e101.

13. Subhi Y, Bube SH, Rolskov Bojsen S, Skou Thomsen AS, Konge L. Expert Involvement and Adherence to Medical Evidence in Medical Mobile Phone Apps: A Systematic Review. *JMIR mHealth uHealth*. 2015;3(3):e79.

14. Fries JF, Spitz P, Kraines RG, Holman HR. Measurement of patient outcome in arthritis. *Arthritis Rheum*. 1980;23(2):137-45.

15. Bruce B, Fries JF. The Stanford Health Assessment Questionnaire: dimensions and practical applications. *Health Qual Life Outcomes*. 2003;1:20.

For peer review only - http://bmjopen.bmj.com/site/about/guidelines.xhtml

16. van Nies JA, Brouwer E, van Gaalen FA, Allaart CF, Huizinga TW, Posthumus MD, et al. Improved early identification of arthritis: evaluating the efficacy of Early Arthritis Recognition Clinics. *Annals of the rheumatic diseases*. 2013;72(8):1295-301.

17. Newsum EC, de Waal MWM, van Steenbergen HW, Gussekloo J, van der Helm-van Mil AHM. How do general practitioners identify inflammatory arthritis? A cohort analysis of Dutch general practitioner electronic medical records. *Rheumatology (Oxford)*. 2016;55(5):848-53.

18. Nederlands Huisartsen Genootschap. NHG-Standaard Artritis (eerste herziening) [Internet]. 2017. https://www.nhg.org/standaarden/volledig/nhg-standaard-artritis (visited on 5 Feb 2019).

19. Ahlmen M, Svensson B, Albertsson K, Forslind K, Hafstrom I. Influence of gender on assessments of disease activity and function in early rheumatoid arthritis in relation to radiographic joint damage. *Ann Rheum Dis*. 2010;69(1):230-3.

20. Krishnan E, Sokka T, Hakkinen A, Hubert H, Hannonen P. Normative values for the Health Assessment Questionnaire disability index: benchmarking disability in the general population. *Arthritis Rheum*. 2004;50(3):953-60.

21. ten Brinck RM, van Dijk BT, van Steenbergen HW, le Cessie S, Numans ME, Hider SL, et al. Development and validation of a clinical rule for recognition of early inflammatory arthritis. *BMJ Open*. 2019;8(11):e023552.

22. White IR, Royston P, Wood AM. Multiple imputation using chained equations: Issues and guidance for practice. *Stat Med*. 2011;30(4):377-99.

23. Marshall A, Altman DG, Holder RL, Royston P. Combining estimates of interest in prognostic modelling studies after multiple imputation: current practice and guidelines. *BMC Med Res Methodol*. 2009;9:57.

24. Bruce B, Fries JF. The Stanford Health Assessment Questionnaire: a review of its history, issues, progress, and documentation. *J Rheumatol*. 2003;30(1):167-78.

25. Bell MJ, Tavares R, Guillemin F, Bykerk VP, Tugwell P, Wells GA. Development of a self-administered early inflammatory arthritis detection tool. *BMC Musculoskelet Disord*. 2010;11:50.

26. Khraishi M, Uphall E, Mong J. The self-administered rheumatoid arthritis (RA) screening questionnaire (RASQ) is a simple and effective tool to detect RA patients. *Ann Rheum Dis*. 2010;69 (suppl 3):374.

27. Maksymowych WP, Russell A, Homik J, Keeling S. E-triage Rheumatoid Arthritis Study in Early Arthritis (ERASE) [Internet]. 2007. http://www.arthritisdoctor.ca/erase-intro.php (visited on 28 March 2019).

28. Singh S, Kumar A, Panneerselvam K, Vennila JJ. Diagnosis of arthritis through fuzzy inference system. *J Med Syst*. 2012;36(3):1459-68.

29. Tavares R, Huang S, Bykerk VP, Bell MJ. A parallel group cohort to determine the measurement properties of an early inflammatory arthritis detection tool. *Rheumatology (Oxford)*. 2013;52(11):2077-85.

30. Almoallim H, Janoudi N, Attar SM, Garout M, Algohary S, Siddiqui MI, et al. Determining early referral criteria for patients with suspected inflammatory arthritis presenting to primary care physicians: a cross-sectional study. *Open Access Rheumatol*. 2017;9:81-90.

For peer review only - http://bmjopen.bmj.com/site/about/guidelines.xhtml

31. Croydon Health Services NHS Foundation Trust. Early arthritis services [Internet]. https://www.croydonhealthservices.nhs.uk/a-to-z-of-services/service/early-arthritis-service-151/ (visited on 19 March 2019).

32. Emery P, Breedveld FC, Dougados M, Kalden JR, Schiff MH, Smolen JS. Early referral recommendation for newly diagnosed rheumatoid arthritis: evidence based development of a clinical guide. *Ann Rheum Dis*. 2002;61(4):290-7.

33. Hampshire Hospitals NHS Foundation Trust. Early Arthritis Clinic referral form [Internet]. https://www.hampshirehospitals.nhs.uk/media/616367/updated_eac_form.pdf (visited on 19 March 2019).

34. Hülsemann J, Mattussek S, Siegmund-Schultze E, Zeidler H. [Early diagnosis of arthritis and spondyloarthritis within the framework of integrated health care in Lower Saxony]. *Z Rheumatol*. 2006;65(1):70-4.

35. Musgrove Park Hospital. Early Arthritis Referral Form [Internet]. https://www.tsft.nhs.uk/media/353049/early-arthritis-final.pdf (visited on 19 March 2019).

36. The National Institute for Health and Care Excellence (NICE). Rheumatoid arthritis in adults: management [Internet]. 2018. https://www.nice.org.uk/guidance/ng100 (visited on 19 March 2019).

For peer review only

For peer review only - http://bmjopen.bmj.com/site/about/guidelines.xhtml

TABLES AND FIGURES

Table 1. Characteristics of patients visiting the Early Arthritis *Recognition* Clinics

	Derivation cohort (n = 997)	1st Validation cohort (n = 506)	2nd Validation cohort (n = 557)
**Inclusion period, years**	2010–2014	2010–2014	2015–2018
**Male, n (%)**	290 (29)	177 (35)	183 (33)
**Age in years, mean ± SD**	51 ± 16	52 ± 16	54 ± 16
**Symptom duration in weeks, median (IQR)**	12 (4–62)	18 (5–69)	13 (4–57)
**Acute onset of symptoms, n (%)**	368 (38)	187 (38)	203 (38)
**Morning stiffness in minutes, median (IQR)**	10 (0–30)	10 (0–31)	10 (0–30)
**Number of painful joints, median (IQR)**	7 (3–15)	9 (4–18)	8 (2–17)
**Number of patient-reported swollen joints, median (IQR)**	2 (1–5)	3 (1–8)	2 (1–5)
**Difficulties with making a fist, n (%)**	242 (47)	130 (54)	291 (54)
**Arthralgia location, n (%)**			
≥1 hand or wrist joint	810 (81)	424 (84)	455 (82)
≥1 forefoot joint	254 (25)	142 (28)	152 (27)
≥1 hand, wrist or forefoot joint	869 (87)	454 (90)	486 (87)
Exclusively large joints*	22 (2)	18 (4)	9 (2)
**HAQ-score, mean ± SD**	0.77 ± 0.59	0.88 ± 0.67	–**
**IA present at joint examination, n (%)**	426 (43)	267 (53)	193 (35)

Legend

Original, unimputed data

* Shoulder, elbow, hip, knee or ankle

** Patients in the second validation cohort did not complete the full HAQ but only the question on difficulties with dressing.

Abbreviations: SD = standard deviation; IQR = interquartile range; IA = inflammatory arthritis.

For peer review only - http://bmjopen.bmj.com/site/about/guidelines.xhtml

Table 2. Association between presence of IA and the degree of difficulties with dressing in the derivation cohort (n = 997), with and without dichotomisation into binary scores

Difficulties with dressing		IA n (%*)	No IA n (%*)	OR (95%CI)	AUC (95%CI)
**Categorical****	0	169 (40)	310 (54)	1.00 (ref)	0.58 (0.55–0.62)
	1	188 (44)	210 (37)	1.64 (1.24–2.16)	
	2	62 (15)	45 (8)	2.49 (1.61–3.84)	
	3	7 (2)	6 (1)	2.20 (0.73–6.66)	
**Binary**	no (0)	169 (40)	310 (54)	1.00 (ref)	0.57 (0.54–0.61)
	yes (≥ 1)	257 (60)	261 (46)	1.80 (1.39–2.33)	

Legend

Results were pooled across 30 imputations; percentages and numbers were rounded.

* Percentage of the total number of patients with and without IA, respectively

** 0 = ‘without any difficulty’; 1 = ‘with some difficulty’; 2 = ‘with much difficulty’; 3 = ‘unable to do’

Abbreviations: IA = inflammatory arthritis; OR = odds ratio; CI = confidence interval; AUC = area under the receiver operating characteristics curve; ref = reference.

For peer review only - http://bmjopen.bmj.com/site/about/guidelines.xhtml

Table 3. Presence of difficulties with dressing associated with presence of IA independently from other clinical variables.

	OR (95%CI) univariable	OR (95%CI) age, gender adjusted	OR (95%CI) multivariable*
**Derivation cohort (n = 997)**	1.80 (1.39–2.33)	1.83 (1.41–2.39)	1.71 (1.27–2.32)
**1st Validation cohort (n = 506)**	2.00 (1.39–2.87)	1.89 (1.31–2.72)	1.64 (1.08–2.50)
**2nd Validation cohort (n = 557)**	2.14 (1.48–3.10)	1.95 (1.32–2.86)	1.87 (1.20–2.92)

Legend

Results were pooled across 30 imputations.

* Adjusted for: being male, age ≥60 years, symptom duration, acuteness of symptom onset, morning stiffness >60 minutes, number of painful joints, presence of patient-reported swollen joint(s) and difficulties with making a fist (see supplementary Table S2 for details).

Abbreviations: IA = inflammatory arthritis; OR = odds ratio; CI = confidence interval.

For peer review only

Figure 1. Observed probabilities of IA according to (A) the total HAQ; (B) the degree of difficulties with dressing; and (C) the presence of difficulties with dressing

IMAGE UPLOADED SEPARATELY

Legend

A. Dot sizes vary according to the number of patients with that particular total HAQ-score. The fit line was calculated by means of binary logistic regression.

Abbreviations: IA = Inflammatory arthritis; HAQ = Health Assessment Questionnaire Disability Index.

For peer review only

Figure 2. Test characteristics of the presence of difficulties with dressing (scores ≥1), with IA at physical examination as outcome

IMAGE UPLOADED SEPARATELY

Legend

Results were pooled across 30 imputations.

Error bars indicate the 95% confidence interval.

Abbreviations: IA = Inflammatory arthritis; Sens. = sensitivity; Spec. = specificity; PPV = positive predictive value; NPV = negative predictive value.

For peer review only - http://bmjopen.bmj.com/site/about/guidelines.xhtml

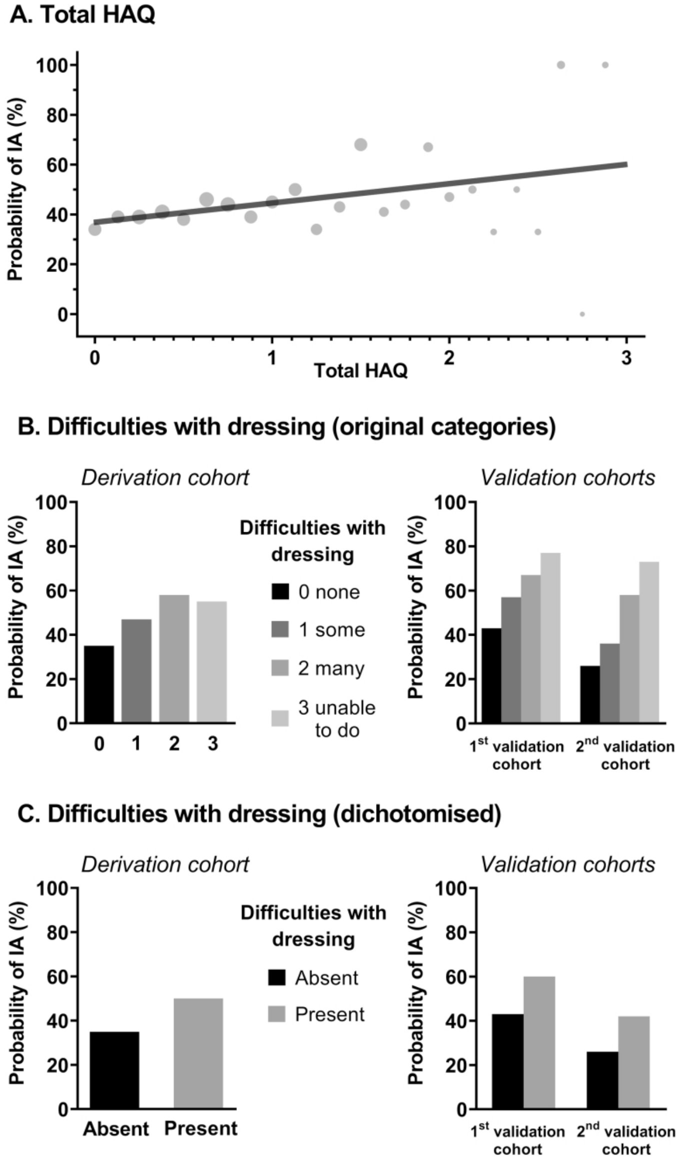

Figure 1. Observed probabilities of IA according to (A) the total HAQ; (B) the degree of difficulties with dressing; and (C) the presence of difficulties with dressing

169x273mm (300 x 300 DPI)

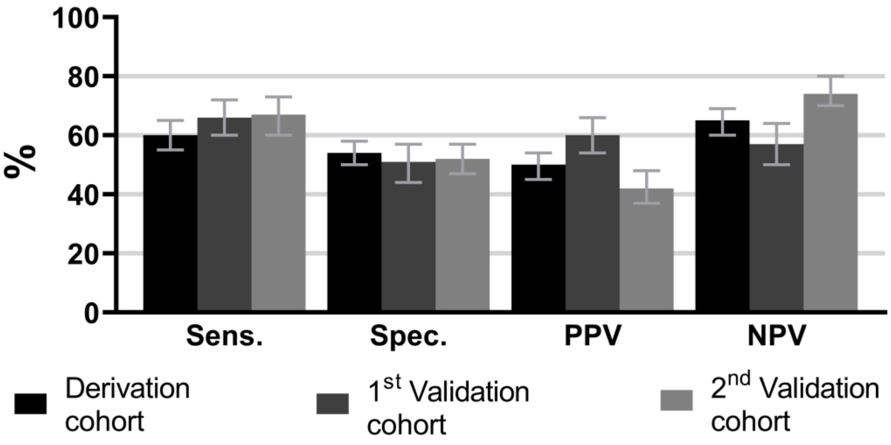

Figure 2. Test characteristics of the presence of difficulties with dressing (scores ≥1), with IA at physical examination as outcome

169x83mm (600 x 600 DPI)

Supplementary file

Table S1. Discriminative ability for the presence of IA of the total HAQ-score and its individual questions in the derivation cohort (n = 997)

Category	Item*	AUC (95%CI)	p
–	Total HAQ-score	0.55 (0.52–0.59)	0.006
**(1) Dressing & grooming**	(A) … Dress yourself, including shoelaces and buttons?	0.58 (0.55–0.62)	<0.001
	(B) … Wash your hair?	0.52 (0.48–0.56)	0.301
**(2) Arising**	(A) … Stand up from a straight chair?	0.55 (0.51–0.58)	0.016
	(B) … Get in and out of bed?	0.53 (0.49–0.57)	0.104
**(3) Eating**	(A) … Cut meat?	0.50 (0.46–0.54)	0.897
	(B) … Lift a full cup or glass to your mouth?	0.51 (0.47–0.55)	0.619
	(C) … Open a new milk carton?	0.48 (0.45–0.52)	0.372
**(4) Walking**	(A) … Walk outdoors on flat ground?	0.57 (0.53–0.60)	0.001
	(B) … Climb up five steps?	0.56 (0.52–0.59)	0.003
**(5) Hygiene**	(A) … Wash and dry your body?	0.54 (0.50–0.58)	0.041
	(B) … Get in and out of a tub bath?	0.53 (0.49–0.57)	0.138
	(C) … Get on and off the toilet?	0.56 (0.52–0.59)	0.003
**(6) Reach**	(A) … Reach and get down a 2½kg object (such as a bag of sugar) from above your head?	0.51 (0.47–0.55)	0.567
	(B) … Bend down to pick up clothing from the floor?	0.52 (0.48–0.56)	0.300
**(7) Grip**	(A) … Open car doors?	0.53 (0.49–0.57)	0.145
	(B) … Open previously opened jars?	0.49 (0.45–0.52)	0.464
	(C) … Turn a faucet on and off?	0.51 (0.47–0.55)	0.514
**(8) Activities**	(A) … Run errands and shop?	0.55 (0.51–0.59)	0.007
	(B) … Get in and out of a car?	0.54 (0.50-0.58)	0.037
	(C) … Do chores such as vacuuming or yard work?	0.55 (0.51–0.59)	0.011

Legend

* Names of categories are shown, as well as individual questions (at the EA*R*C, Dutch translations were used). Each question starts with 'Are you able to …'. Patients indicated the degree of difficulties on each question with 'without any difficulty' (0), 'with some difficulty' (1), 'with much difficulty' (2) or 'unable to do' (3).

Abbreviations: IA = inflammatory arthritis; HAQ = Health Assessment Questionnaire Disability Index; AUC = area under the receiver operating characteristics curve; CI = confidence interval.

For peer review only

Table S2. Presence of difficulties with dressing associated with presence of IA independently from other clinical variables* (detailed overview of multivariable model)

		Derivation cohort (n = 997) OR (95%CI)	1st Validation cohort (n = 506) OR (95%CI)	2nd Validation cohort (n = 557) OR (95%CI)
**Male**		1.99 (1.47–2.70)	1.36 (0.91–2.03)	2.47 (1.66–3.68)
**Age ≥ 60 years**		1.82 (1.35–2.45)	1.40 (0.93–2.10)	1.90 (1.28–2.83)
**Symptom duration in weeks**	≥ 52	1.00 (ref)	1.00 (ref)	1.00 (ref)
	0–5.99	3.63 (2.45–5.39)	1.48 (0.86–2.53)	2.39 (1.38–4.17)
	6–51.99	2.16 (1.50–3.13)	1.24 (0.77–1.99)	1.92 (1.13–3.27)
**Acute onset of symptoms**		0.97 (0.72–1.32)	1.05 (0.69–1.58)	1.19 (0.77–1.83)
**Morning stiffness > 60 minutes**		1.34 (0.84–2.15)	1.59 (0.86–2.93)	0.84 (0.45–1.57)
**Number of painful joints**	0	1.00 (ref)	0.00**	1.00 (ref)
	1–3	4.45 (1.36–14.53)	1.54 (0.95–2.50)	2.70 (0.66–11.00)
	≥ 4	2.34 (0.73–7.53)	1.00 (ref)	1.60 (0.40–6.44)
**Patient-reported swollen joint(s) ≥ 1**		1.97 (1.27–3.04)	1.76 (0.95–3.25)	1.74 (1.07–2.81)
**Difficulties with making a fist**		1.31 (0.92–1.88)	1.54 (0.94–2.54)	1.57 (1.01–2.46)
**Difficulties with dressing**	yes (≥ 1)	1.71 (1.27–2.32)	1.64 (1.08–2.50)	1.87 (1.20–2.92)

Legend

Results were pooled across 30 imputations.

* These variables were selected based on a recent study from the Leiden EA*R*C that determined clinical variables that associated with the presence of IA.

** In the Groningen validation cohort none of the patients who indicated no painful joints on the questionnaire were positive for IA upon joint examination. Therefore, no meaningful CI could be calculated for this group and patients with ≥4 painful joints were assigned as reference group instead.

Abbreviations: IA = inflammatory arthritis; OR = odds ratio; CI = confidence interval, ref = reference.

For peer review only

Table S3. Characteristics of patients with and without available data on difficulties with dressing

	Derivation cohort			1st Validation cohort			2nd Validation cohort		
Data on difficulties with dressing:	Available (n = 956)	Missing (n = 41)	p*	Available (n = 495)	Missing (n = 11)	p*	Available (n = 542)	Missing (n = 15)	p*
Male, n (%)	275 (29)	15 (37)	0.294	174 (35)	3 (27)	0.755	180 (33)	3 (20)	0.406
Age in years, mean ± SD	51 ± 16	57 ± 17	0.015	51 ± 16	57 ± 10	0.237	54 ± 16	58 ± 12	0.312
Symptom duration in weeks, median (IQR)	13 (4–62)	9 (3–72)	0.792	18 (5–69)	7 (5–64)	0.519	13 (4–59)	13 (5–24)	0.750
Acute onset of symptoms, n (%)	353 (38)	15 (41)	0.731	185 (38)	2 (20)	0.333	201 (37)	2 (29)	0.713
Morning stiffness in minutes, median (IQR)	10 (0–30)	4 (0–60)	0.983	10 (0–33)	10 (1–190)	0.776	10 (0–30)	0 (0–1)	0.012
Number of painful joints, median (IQR)	7 (3–14)	6 (2–25)	0.941	9 (4–18)	9 (3–20)	0.840	8 (3–17)	1 (1–9)	0.021
Number of patient-reported swollen joints, median (IQR)	2 (1–5)	2 (0–5)	0.503	3 (1–8)	5 (2–8)	0.698	2 (1–5)	1 (0–5)	0.317
Difficulties with making a fist, n (%)	239 (48)	3 (23)	0.095	128 (54)	2 (50)	1.000	288 (55)	3 (50)	1.000

Legend

Percentages are based on available questionnaire entries.

* Unpaired t-tests, Fisher's Exact tests and Mann-Whitney U tests were used as appropriate.

Abbreviations: SD = standard deviation; IQR = interquartile range.

For peer review only

Table S4. (sensitivity analyses limited to unimputed data) Association between presence of difficulties with dressing and presence of IA in the derivation and validation cohorts

	OR (95%CI) univariable	OR (95%CI) age, gender adjusted	OR (95%CI) multivariable*
**Derivation cohort**	1.83 (1.41–2.38)	1.87 (1.43–2.44)	1.59 (0.98–2.57)
**1st Validation cohort**	2.02 (1.41–2.91)	1.92 (1.33–2.77)	1.70 (0.83–3.84)
**2nd Validation cohort**	2.19 (1.51–3.17)	1.99 (1.36–2.93)	1.91 (1.16–3.16)

Legend

* Point estimates are similar to those obtained in the main analyses, but have broader confidence intervals since only complete cases could be analysed. Covariates: being male, age ≥60 years, symptom duration, acuteness of symptom onset, morning stiffness >60 minutes, number of painful joints, presence of patient-reported swollen joint(s) and difficulties with making a fist.

Abbreviations: IA = inflammatory arthritis; OR = odds ratio; CI = confidence interval.

For peer review only

STROBE 2007 (v4) Statement—Checklist of items that should be included in reports of *cross-sectional studies*

Section/Topic	Item #	Recommendation	Reported on page #
**Title and abstract**	1	(*a*) Indicate the study's design with a commonly used term in the title or the abstract	1, 2
		(*b*) Provide in the abstract an informative and balanced summary of what was done and what was found	2
**Introduction**			
Background/rationale	2	Explain the scientific background and rationale for the investigation being reported	5, 6
Objectives	3	State specific objectives, including any prespecified hypotheses	6
**Methods**			
Study design	4	Present key elements of study design early in the paper	6–8
Setting	5	Describe the setting, locations, and relevant dates, including periods of recruitment, exposure, follow-up, and data collection	6–8
Participants	6	(*a*) Give the eligibility criteria, and the sources and methods of selection of participants	6, 7
Variables	7	Clearly define all outcomes, exposures, predictors, potential confounders, and effect modifiers. Give diagnostic criteria, if applicable	8, 9
Data sources/ measurement	8*	For each variable of interest, give sources of data and details of methods of assessment (measurement). Describe comparability of assessment methods if there is more than one group	7
Bias	9	Describe any efforts to address potential sources of bias	9, 10
Study size	10	Explain how the study size was arrived at	7
Quantitative variables	11	Explain how quantitative variables were handled in the analyses. If applicable, describe which groupings were chosen and why	8, 9
Statistical methods	12	(*a*) Describe all statistical methods, including those used to control for confounding	9, 10
		(*b*) Describe any methods used to examine subgroups and interactions	*NA*
		(*c*) Explain how missing data were addressed	9, 10
		(*d*) If applicable, describe analytical methods taking account of sampling strategy	*NA*
		(*e*) Describe any sensitivity analyses	10
**Results**			

Participants	13*	(a) Report numbers of individuals at each stage of study—eg numbers potentially eligible, examined for eligibility, confirmed eligible, included in the study, completing follow-up, and analysed	21
		(b) Give reasons for non-participation at each stage	7
		(c) Consider use of a flow diagram	*NA*
Descriptive data	14*	(a) Give characteristics of study participants (eg demographic, clinical, social) and information on exposures and potential confounders	21
		(b) Indicate number of participants with missing data for each variable of interest	9
Outcome data	15*	Report numbers of outcome events or summary measures	10–12, 21–23
Main results	16	(*a*) Give unadjusted estimates and, if applicable, confounder-adjusted estimates and their precision (eg, 95% confidence interval). Make clear which confounders were adjusted for and why they were included	23
		(*b*) Report category boundaries when continuous variables were categorized	22
		(*c*) If relevant, consider translating estimates of relative risk into absolute risk for a meaningful time period	*NA*
Other analyses	17	Report other analyses done—eg analyses of subgroups and interactions, and sensitivity analyses	12
**Discussion**			
Key results	18	Summarise key results with reference to study objectives	13
Limitations	19	Discuss limitations of the study, taking into account sources of potential bias or imprecision. Discuss both direction and magnitude of any potential bias	15
Interpretation	20	Give a cautious overall interpretation of results considering objectives, limitations, multiplicity of analyses, results from similar studies, and other relevant evidence	16
Generalisability	21	Discuss the generalisability (external validity) of the study results	15, 16
**Other information**			
Funding	22	Give the source of funding and the role of the funders for the present study and, if applicable, for the original study on which the present article is based	17

*Give information separately for cases and controls in case-control studies and, if applicable, for exposed and unexposed groups in cohort and cross-sectional studies.

Note: An Explanation and Elaboration article discusses each checklist item and gives methodological background and published examples of transparent reporting. The STROBE checklist is best used in conjunction with this article (freely available on the Web sites of PLoS Medicine at http://www.plosmedicine.org/, Annals of Internal Medicine at http://www.annals.org/, and Epidemiology at http://www.epidem.com/). Information on the STROBE Initiative is available at www.strobe-statement.org.

For peer review only

Section & Topic	No	Item	Reported on page #
**TITLE OR ABSTRACT**			
	**1**	Identification as a study of diagnostic accuracy using at least one measure of accuracy (such as sensitivity, specificity, predictive values, or AUC)	1–2
**ABSTRACT**			
	**2**	Structured summary of study design, methods, results, and conclusions (for specific guidance, see STARD for Abstracts)	2
**INTRODUCTION**			
	**3**	Scientific and clinical background, including the intended use and clinical role of the index test	5–6
	**4**	Study objectives and hypotheses	6
**METHODS**			
Study design	**5**	Whether data collection was planned before the index test and reference standard were performed (prospective study) or after (retrospective study)	*NA (differed per cohort; see p. 8 under 'HAQ')*
Participants	**6**	Eligibility criteria	6–7
	**7**	On what basis potentially eligible participants were identified (such as symptoms, results from previous tests, inclusion in registry)	6–7
	**8**	Where and when potentially eligible participants were identified (setting, location and dates)	6–7
	**9**	Whether participants formed a consecutive, random or convenience series	2, 7
Test methods	**10a**	Index test, in sufficient detail to allow replication	8
	**10b**	Reference standard, in sufficient detail to allow replication	8
	**11**	Rationale for choosing the reference standard (if alternatives exist)	8
	**12a**	Definition of and rationale for test positivity cut-offs or result categories of the index test, distinguishing pre-specified from exploratory	9
	**12b**	Definition of and rationale for test positivity cut-offs or result categories of the reference standard, distinguishing pre-specified from exploratory	*NA*
	**13a**	Whether clinical information and reference standard results were available to the performers/readers of the index test	8
	**13b**	Whether clinical information and index test results were available to the assessors of the reference standard	8
Analysis	**14**	Methods for estimating or comparing measures of diagnostic accuracy	9
	**15**	How indeterminate index test or reference standard results were handled	*NA*
	**16**	How missing data on the index test and reference standard were handled	9–10
	**17**	Any analyses of variability in diagnostic accuracy, distinguishing pre-specified from exploratory	15
	**18**	Intended sample size and how it was determined	6–7
**RESULTS**			
Participants	**19**	Flow of participants, using a diagram	8 (diagram *NA* because of simplicity)
	**20**	Baseline demographic and clinical characteristics of participants	10, 22
	**21a**	Distribution of severity of disease in those with the target condition	*NA* (see p. 8/16)
	**21b**	Distribution of alternative diagnoses in those without the target condition	*NA* (see p. 8/16)
	**22**	Time interval and any clinical interventions between index test and reference standard	*NA* (same visit)
Test results	**23**	Cross tabulation of the index test results (or their distribution) by the results of the reference standard	22
	**24**	Estimates of diagnostic accuracy and their precision (such as 95% confidence intervals)	10–12
	**25**	Any adverse events from performing the index test or the reference standard	*NA*
**DISCUSSION**			
	**26**	Study limitations, including sources of potential bias, statistical uncertainty, and generalisability	15–16
	**27**	Implications for practice, including the intended use and clinical role of the index test	16
**OTHER INFORMATION**			
	**28**	Registration number and name of registry	*NA*
	**29**	Where the full study protocol can be accessed	*NA*

For peer review only - http://bmjopen.bmj.com/site/about/guidelines.xhtml

	**30**	Sources of funding and other support; role of funders	17

For peer review only

STARD 2015

AIM

STARD stands for "Standards for Reporting Diagnostic accuracy studies". This list of items was developed to contribute to the completeness and transparency of reporting of diagnostic accuracy studies. Authors can use the list to write informative study reports. Editors and peer-reviewers can use it to evaluate whether the information has been included in manuscripts submitted for publication.

EXPLANATION

A **diagnostic accuracy study** evaluates the ability of one or more medical tests to correctly classify study participants as having a **target condition.** This can be a disease, a disease stage, response or benefit from therapy, or an event or condition in the future. A medical test can be an imaging procedure, a laboratory test, elements from history and physical examination, a combination of these, or any other method for collecting information about the current health status of a patient.

The test whose accuracy is evaluated is called **index test.** A study can evaluate the accuracy of one or more index tests. Evaluating the ability of a medical test to correctly classify patients is typically done by comparing the distribution of the index test results with those of the **reference standard**. The reference standard is the best available method for establishing the presence or absence of the target condition. An accuracy study can rely on one or more reference standards.

If test results are categorized as either positive or negative, the cross tabulation of the index test results against those of the reference standard can be used to estimate the **sensitivity** of the index test (the proportion of participants *with* the target condition who have a positive index test), and its **specificity** (the proportion *without* the target condition who have a negative index test). From this cross tabulation (sometimes referred to as the contingency or "2x2" table), several other accuracy statistics can be estimated, such as the positive and negative **predictive values** of the test. Confidence intervals around estimates of accuracy can then be calculated to quantify the statistical **precision** of the measurements.

If the index test results can take more than two values, categorization of test results as positive or negative requires a **test positivity cut-off**. When multiple such cut-offs can be defined, authors can report a receiver operating characteristic (ROC) curve which graphically represents the combination of sensitivity and specificity for each possible test positivity cut-off. The **area under the ROC curve** informs in a single numerical value about the overall diagnostic accuracy of the index test.

The **intended use** of a medical test can be diagnosis, screening, staging, monitoring, surveillance, prediction or prognosis. The **clinical role** of a test explains its position relative to existing tests in the clinical pathway. A replacement test, for example, replaces an existing test. A triage test is used before an existing test; an add-on test is used after an existing test.

Besides diagnostic accuracy, several other outcomes and statistics may be relevant in the evaluation of medical tests. Medical tests can also be used to classify patients for purposes other than diagnosis, such as staging or prognosis. The STARD list was not explicitly developed for these other outcomes, statistics, and study types, although most STARD items would still apply.

DEVELOPMENT

This STARD list was released in 2015. The 30 items were identified by an international expert group of methodologists, researchers, and editors. The guiding principle in the development of STARD was to select items that, when reported, would help readers to judge the potential for bias in the study, to appraise the applicability of the study findings and the validity of conclusions and recommendations. The list represents an update of the first version, which was published in 2003.

More information can be found on http://www.equator-network.org/reporting-guidelines/stard.

For peer review only

CC BY SA

BMJ Open

The value of inquiring about functional impairments for early identification of inflammatory arthritis: a large cross-sectional derivation and validation study from the Netherlands

Journal:	*BMJ Open*
Manuscript ID	bmjopen-2020-040148.R2
Article Type:	Original research
Date Submitted by the Author:	07-Sep-2020
Complete List of Authors:	van Dijk, Bastiaan; Leiden University Medical Center,<br>van Steenbergen, Hanna; Leiden University Medical Center, Rheumatology<br>Niemantsverdriet, Ellis; Leiden University Medical Center, Rheumatology<br>Brouwer, Elisabeth; University Medical Centre Groningen, Rheumatology and Clinical Immunology<br>van der Helm-van Mil, Annette; Leiden University Medical Center, Rheumatology; Erasmus Medical Center, Rheumatology
**Primary Subject Heading**:	Rheumatology
Secondary Subject Heading:	General practice / Family practice
Keywords:	RHEUMATOLOGY, PRIMARY CARE, Rheumatology < INTERNAL MEDICINE

SCHOLARONE™
Manuscripts

I, the Submitting Author has the right to grant and does grant on behalf of all authors of the Work (as defined in the below author licence), an exclusive licence and/or a non-exclusive licence for contributions from authors who are: i) UK Crown employees; ii) where BMJ has agreed a CC-BY licence shall apply, and/or iii) in accordance with the terms applicable for US Federal Government officers or employees acting as part of their official duties; on a worldwide, perpetual, irrevocable, royalty-free basis to BMJ Publishing Group Ltd ("BMJ") its licensees and where the relevant Journal is co-owned by BMJ to the co-owners of the Journal, to publish the Work in this journal and any other BMJ products and to exploit all rights, as set out in our licence.

The Submitting Author accepts and understands that any supply made under these terms is made by BMJ to the Submitting Author unless you are acting as an employee on behalf of your employer or a postgraduate student of an affiliated institution which is paying any applicable article publishing charge ("APC") for Open Access articles. Where the Submitting Author wishes to make the Work available on an Open Access basis (and intends to pay the relevant APC), the terms of reuse of such Open Access shall be governed by a Creative Commons licence – details of these licences and which Creative Commons licence will apply to this Work are set out in our licence referred to above.

Other than as permitted in any relevant BMJ Author's Self Archiving Policies, I confirm this Work has not been accepted for publication elsewhere, is not being considered for publication elsewhere and does not duplicate material already published. I confirm all authors consent to publication of this Work and authorise the granting of this licence.

For peer review only

Original research

The value of inquiring about functional impairments for early identification of inflammatory arthritis: a large cross-sectional derivation and validation study from the Netherlands

For peer review only

Bastiaan T. van Dijk[1], Hanna W. van Steenbergen[1], Ellis Niemantsverdriet[1], Elisabeth Brouwer[2], Annette H.M. van der Helm-van Mil[1,3]

[1] Department of Rheumatology, Leiden University Medical Centre, Leiden, the Netherlands

[2] Department of Rheumatology and Clinical Immunology, University Medical Centre Groningen, Groningen, the Netherlands

[3] Department of Rheumatology, Erasmus Medical Centre, Rotterdam, the Netherlands

Corresponding authors

(1) B.T. van Dijk, M.D., Department of Rheumatology, Leiden University Medical Centre

P.O. Box 9600, 2300 RC Leiden, the Netherlands

Tel.: +31 71 5265655; Email: B.T.van_Dijk@lumc.nl

ORCID ID: 0000-0002-5161-6791

(2) A.H.M. (Annette) van der Helm-van Mil, M.D./PhD/professor, Departments of Rheumatology, Leiden University Medical Centre and Erasmus Medical Centre

PO Box 2040, 3000 CA Rotterdam, the Netherlands

Tel.: +31 71 5265655; Email: a.h.m.vanderhelm@erasmusmc.nl

ORCID ID: 0000-0001-8572-1437

For peer review only - http://bmjopen.bmj.com/site/about/guidelines.xhtml

For peer review only

For peer review only - http://bmjopen.bmj.com/site/about/guidelines.xhtml

ABSTRACT

Objectives. Healthcare professionals other than rheumatologists experience difficulties in detecting early inflammatory arthritis (IA) by joint examination. Self-reported symptoms are increasingly considered as helpful and could be incorporated in online tools to assist healthcare professionals, but firstly their discriminative ability must be assessed. As part of this effort, we evaluated whether inquiring about functional impairments could aid early IA-identification.

Design. Cross-sectional derivation and validation study.

Setting. Data from two Early Arthritis *Recognition* Clinics (EA*R*C) in the Netherlands were studied, which are easy-access outpatient rheumatology clinics intermediary between primary and secondary care for patients in whom GPs suspect but are unsure about IA-presence.

Participants. Between 2010-2014, 997 patients consecutively visited the Leiden-EA*R*C (derivation cohort). Patients consecutively visiting the Groningen-EA*R*C (2010-2014, n=506) and Leiden-EA*R*C (2015-2018, n=557) served as validation cohorts.

Primary and secondary outcome measures. Physical functioning was assessed with the Health Assessment Questionnaire Disability-Index (HAQ); IA-presence by physical joint examination by rheumatologists. HAQ-questions were studied individually regarding discriminative ability for IA-presence. For the best discriminating question, odds ratios (ORs) and positive predictive values (PPVs) for IA-presence were determined.

Results. IA was ascertained in 43% (derivation cohort), 53% and 35% (validation cohorts). In the derivation cohort, IA-presence associated with higher mean HAQ-scores (0.84 versus 0.73, p=0.003). One question on difficulties with dressing equalled discriminative ability of the total HAQ-score. 'Difficulties with dressing' yielded ORs for IA-presence of 1.8(95%CI

For peer review only - http://bmjopen.bmj.com/site/about/guidelines.xhtml

1.4-2.4) in the derivation cohort; 2.0(1.4-2.9) and 2.1(1.5-3.1) in the validation cohorts. After adjustments for clinical characteristics these were 1.7(1.3-2.3), 1.6(1.1-2.5) and 1.9(1.2-2.9). PPVs (probabilities of IA for positive answers) ranged 42-60% and NPVs (probabilities of no IA for negative answers) ranged 57-74%.

Conclusions. Patient-reported difficulties with dressing in patients with suspected IA associated with actual IA-presence. Although further validation is required, e.g. in primary care, this simple question could be of help in future early IA-detection tools for healthcare professionals with limited experience in joint examination.

For peer review only

For peer review only - http://bmjopen.bmj.com/site/about/guidelines.xhtml

STRENGHTS AND LIMITATIONS OF THIS STUDY

- We evaluated the value of questions about functional impairments for early recognition of inflammatory arthritis (IA), for which new and simple tools are urgently needed – especially in primary care.
- This was studied in a setting intermediary between primary and secondary care that services patients whose general practitioners (GPs) were doubtful about the presence of IA; the population for whom such new tools are most relevant.
- This association was tested for independence from known predictors and validated in two independent cohorts.
- However, further validation in primary care is needed, since data were not yet collected in actual primary care practices.

For peer review only

INTRODUCTION

In rheumatoid arthritis (RA), early initiation of treatment with disease-modifying antirheumatic drugs (DMARDs) is associated with improved outcomes.[1,2] Also for other types of inflammatory arthritis (IA) the importance of early treatment is increasingly recognised.[3-5] To achieve early treatment initiation however, it is necessary for at-risk patients to be referred to a rheumatologist in a timely fashion.[6]

Consequently, general practitioners (GPs) play a crucial role in early identification of IA, but they have indicated considerable barriers regarding timely and adequate referral of patients with musculoskeletal complaints, including a lack of self-confidence in detecting synovitis.[7] GPs generally have limited experience with the detection of IA by joint palpation compared to rheumatologists, since their patient population is characterised by a relatively low incidence of RA and IA (0.7/1000 patient years in the Netherlands, which amounts to 1–2 new patients on average per GP per year) compared to a much higher incidence of other musculoskeletal complaints.[8-10] These circumstances may add to referral delay at the level of the initial healthcare provider (usually the GP), which was found to be an important contributor to overall delay until treatment initiation in many European countries.[11]

In this light, the search for novel strategies to improve early recognition and referral of patients at risk for IA and RA remains of great importance. This was emphasised in the latest update of the European League against Rheumatism (EULAR) research agenda for early arthritis by including 'the identification of tools that could help GPs diagnose early arthritis and prioritise referral.'[6] Given the increasing usage of healthcare-related mobile applications (apps)[12], new tools are preferred to be simple and time-efficient, providing suitability for possible future digital initiatives for early IA-screening. In addition to ease-of-

For peer review only - http://bmjopen.bmj.com/site/about/guidelines.xhtml

use, such tools should be based on valid and reliable data but currently most medical mobile apps are not properly evidence-based, highlighting the need for thorough research.[13]

Patients with IA generally experience limitations in physical functioning. One could hypothesise that these limitations may differ in sort and severity compared to those related to other, less urgent causes of joint symptoms. Functional impairments in patients with rheumatic diseases are generally assessed using the Health Assessment Questionnaire Disability-Index (HAQ),[14] but use of this patient-reported outcome instrument is mostly limited to research in established rheumatic disease instead of earlier stages. Nonetheless, it is generally considered a self-explanatory and relatively efficient questionnaire (it can be completed within five minutes).[15] In order to search for tools that might facilitate early identification of IA we performed a cross-sectional study on the association between functional impairments (measured by the HAQ) and presence of IA at joint examination (identified by rheumatologists). Aiming for simple and time-efficient use in daily practice, we also studied the HAQ-questions separately and evaluated whether a single question could facilitate early identification of IA. Although information on functional impairments alone is not expected to have sufficient accuracy, it may be used as part of a more comprehensive tool that could be incorporated in a screening app for early IA. Findings were verified in two validation cohorts.

For peer review only

PATIENTS AND METHODS

Patients

Aiming to decrease referral delay in early IA, Early Arthritis *Recognition* Clinics (EA*R*C) were initiated in 2010 at the Leiden and Groningen University Medical Centres (LUMC and UMCG) in the Netherlands. The EA*R*C design has been described previously.[16] In short,

For peer review only - http://bmjopen.bmj.com/site/about/guidelines.xhtml

EA*R*Cs are clinics for patients in whom their GP suspects but is unsure about the presence of IA. These easy-access clinics are characterised by their unique intermediate setting, namely in between primary and secondary care. The EA*R*Cs have no waiting lists, can be visited by referred patients once to twice a week without an appointment and have markedly reduced referral delay.[16] GPs in the region are instructed to quickly refer any patient for whom they are unsure about the presence of IA, instead of applying a strategy of 'watchful waiting' or performing additional diagnostic tests. Notably, auto-antibodies or other additional investigations are generally not performed by GPs in our region[17], which is in accordance with the Dutch national guidelines.[18]

At the EA*R*C, patients completed a brief clinical questionnaire on their symptoms, followed by a short consultation and full 66-joint examination by an experienced rheumatologist. If IA was detected at physical examination (by palpable joint swelling), patients were analysed at the regular rheumatology outpatient clinic for further diagnosis. Otherwise, they were referred back to their GP. Laboratory tests and imaging were not performed at the EA*R*C.

The derivation cohort comprised 997 patients who consecutively visited the Leiden-EA*R*C between September 2010 and April 2014. The first validation cohort consisted of 506 patients who visited the Groningen-EA*R*C from September 2010 to January 2014. The second validation cohort consisted of 557 patients who visited the Leiden-EA*R*C from November 2015 to December 2018. Patients who visited the Leiden-EA*R*C from April 2014 to November 2015 did not fill out questions on physical functioning and were not included in this study.

For peer review only - http://bmjopen.bmj.com/site/about/guidelines.xhtml

Patient and Public involvement

Patient research partners agreed with the pathway of care at EA*R*C and provided feedback on the questionnaire, which was expanded in 2012 with two questions.

HAQ

Physical functioning was assessed during the same EA*R*C-visit using the HAQ, a questionnaire containing 20 questions on impairments across eight functional categories.[14,15] Each question was scored by patients on a 4-point scale ranging 0–3, representing the degree of difficulties experienced when performing the corresponding activity, with 0 indicating no difficulties and 3 indicating full disability. The total HAQ score is calculated as the average across eight categories and ranges 0–3. Patients in the derivation cohort and patients in the first validation cohort filled out the full HAQ. Based on initial results from the derivation cohort, patients in the second validation cohort were asked to complete only question 1A on difficulties with dressing.

Outcome

The outcome used in all statistical analyses was presence of IA, determined by the rheumatologist at physical examination. Final classifying diagnoses were made during subsequent visit(s) at the regular rheumatology outpatient clinic and were beyond the scope of this study. The rheumatologists who determined IA at joint examination were not formally blinded to the HAQ-data; at request however the participating rheumatologists answered not to consider HAQ-data in their evaluation of the presence of IA (personal communication).

For peer review only - http://bmjopen.bmj.com/site/about/guidelines.xhtml

Statistical analyses

In the derivation cohort, total HAQ-scores were compared between patients with and without IA at joint examination. Areas under the curve (AUCs) were determined for the total HAQ-score as well as its questions individually, in order to select the best discriminating question for further investigation (based on highest AUC). The use of aids and devices was taken into account for calculation of the total HAQ-score as prescribed, but not for scores on individual questions.[14,15] For the best discriminating question, odds ratios (ORs) and AUCs were determined with both the original, categorical responses and with binary responses (no vs. any degree of difficulties; i.e. scores ≥1), since a binary tool would be even more suited for easy use in daily practice. ORs were adjusted for age and gender.[19-21] Test characteristics and probabilities of IA were calculated. Analyses were repeated in the two validation cohorts.

A previous study from the Leiden-EA*R*C derived an algorithm for IA-identification, consisting of clinical variables that were independently associated with the presence of IA (being male, age ≥60 years, symptom duration, acuteness of symptom onset, morning stiffness >60 minutes, number of painful joints, presence of patient-reported swollen joint(s) and difficulties with making a fist).[21] These variables were included in multivariable logistic regression analyses, to study if self-reported functional disabilities were independently associated with IA. In addition, change in diagnostic accuracy when adding functional disability to the algorithm was assessed in the derivation cohort by comparing AUCs.

The HAQ-question of interest was missing in 4%, 2% and 3% of the cohorts respectively. Characteristics of patients with available and missing data on this HAQ-question were compared. Missing values were imputed using multiple imputation by chained equations (MICE) with predictive mean matching (PMM).[22] Rubin's rules were applied to pool point estimates and confidence intervals across 30 imputations.[23] Imputed data were used for the

For peer review only - http://bmjopen.bmj.com/site/about/guidelines.xhtml

main analyses. In sensitivity analyses, results were compared to those obtained from the unimputed data.

IBM SPSS (version 25) and R (version 3.5.2) were used. P-values <0.05 were considered statistically significant.

RESULTS

Patients

Patient characteristics for all three cohorts are presented in Table 1. Over 80% in each cohort presented with hand and/or wrist arthralgia. IA was diagnosed in 43%, 53% and 35% of patients, respectively. Patients in the first validation cohort had a slightly longer symptom duration (median 18 weeks) than patients in the derivation and second validation cohorts (median 12 and 13 weeks, respectively). All other characteristics were similar.

Discriminative ability of a single question equalled the total HAQ score

Patients with IA at physical examination had a higher mean total HAQ score than patients without IA in the derivation cohort (0.84 (95%CI 0.77–0.89) compared to 0.73 (0.67–0.77), p=0.003), indicating a higher degree of functional disability. A gradual increase of the probability of IA at joint examination was observed as total HAQ-scores rose (Figure 1A).

To search for a method that is more simple and time-efficient for usage in daily practice than the 20-question total HAQ, its questions were also studied individually (supplementary Table S1). Of these questions, scores on ‘difficulties with dressing’ (question 1A) yielded the highest AUC, which equalled the AUC of the total HAQ score: 0.58 (0.55–0.62) vs. 0.55 (0.52–0.59), respectively. This indicates that, in addition to greater ease of use, the

For peer review only - http://bmjopen.bmj.com/site/about/guidelines.xhtml

For peer review only

discriminative ability of just one question was equal to the total HAQ. For these reasons, this question (“Are you able to dress yourself, including shoelaces and buttons?”) was considered the best discriminating question and was studied further instead of the total HAQ-score. The percentage of patients with different categories of impairment among the patients with and without IA are presented in Table 2. The probabilities of IA-presence for the different categories of impairments with dressing are indicated in Figure 1B; the probability increased with more impairment.

Since a binary question is even easier to implement in practice, we assessed the discriminative ability of dichotomised scores on difficulties with dressing. Loss of overall discriminative ability, measured using the AUC, induced by dichotomisation into a binary score was minor (AUC of 0.57 (0.54–0.61) vs. 0.58 (0.55–0.62)). Observed probabilities of IA-presence with this dichotomised score are presented in Figure 1C. For the benefit of ease of use this minor difference was accepted and dichotomised scores were used in subsequent analyses.

Difficulties with dressing were independently associated with presence of IA in derivation cohort

ORs for the IA-presence of a confirmative answer on the question about difficulties with dressing are presented in Table 3. In the derivation cohort, the presence of difficulties with dressing (scores ≥1) was associated with presence of IA with an age and gender adjusted OR of 1.83 (1.41–2.39). After further adjustment for the aforementioned clinical variables that were previously identified as associated with IA (being male, age ≥60 years, symptom duration, acuteness of symptom onset, morning stiffness >60 minutes, number of painful joints, presence of patient-reported swollen joint(s) and difficulties with making a fist), difficulties with dressing remained independently associated with the presence of IA, with an

For peer review only - http://bmjopen.bmj.com/site/about/guidelines.xhtml

OR of 1.71 (1.27–2.32). ORs for the other clinical variables are provided in the supplementary file (supplementary Table S2). The AUC of the model with only the aforementioned clinical variables was 0.72 (0.69–0.76), while the model where difficulties with dressing was added had an AUC of 0.73 (0.70–0.77). Since the presence of difficulties with dressing was independently associated with IA it was subsequently studied in the validation cohorts.

Difficulties with dressing were independently associated with presence of IA in both validation cohorts

In the first and second validation cohorts, age and gender adjusted ORs were 1.89 (1.31–2.72) and 1.95 (1.32–2.86), respectively. After further adjustment for the aforementioned clinical variables that were previously identified as associated with IA, ORs were 1.64 (1.08–2.50) and 1.87 (1.20–2.92), respectively. Thus, the independent association of difficulties with dressing with presence of IA was conformed in both validation cohorts.

Predictive values and test characteristics

Lastly, we studied test characteristics and predictive values of positive answers on just the single question about difficulties with dressing (Figure 2). The negative predictive values (NPVs) in the different cohorts ranged between 57–74%, indicating that 57–74% of patients without difficulties with dressing had no IA at physical examination. The positive predictive values (PPVs) ranged between 42–60%, meaning that 42–60% of patients who reported to have difficulties with dressing were indeed identified as having IA.

For peer review only

For peer review only - http://bmjopen.bmj.com/site/about/guidelines.xhtml

Sensitivity analyses on unimputed data

No clinically relevant differences in characteristics of patients with and without information on 'difficulties with dressing' were found (supplementary Table S3). Sub-analyses limited to the unimputed data (supplementary Table S4) yielded similar results as in the main analyses.

DISCUSSION

Healthcare professionals who are not rheumatologists generally have limited experience in joint examination, which hampers them in early identification of IA. For GPs this contributes to referral delay and necessitates the development of feasible tools that could support early identification of IA. We investigated the value of information on functional impairments in a cross-sectional study including patients with suspected but doubtful early IA according to GPs. We observed that, among patients with suspected IA according to GPs, those with actual IA according to the rheumatologist had higher HAQ-scores, and that the discriminative ability of a binary answer to a single question on difficulties with dressing was equal to that of the total HAQ. Results were validated in independent cohorts of a similar setting, namely in between primary and secondary care. Together the results imply that easily obtainable information on functional impairments may be of value in facilitating early detection of IA.

The HAQ was originally designed for patient-reported outcome assessment and not as a diagnostic tool.[14] However, it is the most widely used and validated instrument in the field of rheumatology for measuring functional impairments.[15,24] The HAQ was neither designed to use in parts (and assess questions individually) when used as outcome measure. Despite these limitations, the current data suggest that a simple question on difficulties with dressing, as deduced from the total HAQ, is of discriminative relevance. The simplicity of a single question and binary answers (no vs. any difficulties with dressing) may facilitate the

For peer review only - http://bmjopen.bmj.com/site/about/guidelines.xhtml

use of assessing functional impairments for diagnostic purposes in busy daily practice and as part of future internet or mobile app based tools for early identification of IA.

The best discriminating question was one related to hand function. Interestingly, over 80% of patients reported to have symptoms of the hands (Table 1). Presumably, GPs are more often doubtful about IA in hand joints since these are relatively small joints and synovitis is often subtle. Alternatively, GPs may be more inclined to assess hand joints compared to other joints. Exploratory subgroup analyses limited to patients who indicated to have hand symptoms yielded similar results (data not shown).

The current study was, to the best of our knowledge, the first to investigate the discriminative ability of functional impairments measured by the HAQ in the context of identifying early IA. Several other tools to promote detection of early IA have been reported, including self-administered questionnaires and internet-based tools for primary care providers.[25-28] However, none of these were validated tools that incorporated patient-reported functional impairments. The best studied initiative, the Early Inflammatory Arthritis Detection Tool reported by Bell MJ et al,[25] contained one question on functional impairments in general, namely whether important activities were affected by joint problems. Although this tool showed good overall discriminative ability, it was externally validated only in a tertiary care setting.[29] Its usability in cases of diagnostic uncertainty remains undetermined.

It is unknown how patient-reported functional impairments currently influence referral policy in patients with suspected IA in primary care. Information on functional limitations is generally lacking in local guidelines and referral criteria.[18,30-36] The Dutch GP guideline on IA, for example, recommends asking patients about the impact of their joint complaints on their daily lives, but does not specify the information that should be collected and in which way this should be incorporated into decision-making and referral policy.[18]

For peer review only - http://bmjopen.bmj.com/site/about/guidelines.xhtml

For peer review only

Although we attempted to seek for evidence on simple methods that are helpful to identify IA, the AUC reached by 'difficulties with dressing' was fairly limited. It is not surprising that a single question did not perform equally accurate as the reference, i.e. evaluation of swollen joints with joint palpation. However, particularly in the current absence of other evidence-based strategies, this simple question that requires almost no extra effort, time or resources may be of value in improving early identification of IA.

The main strengths of the current study are related to the unique easy-access setting of the EA*R*Cs intermediary between primary and secondary care. Firstly, the EA*R*Cs could be visited weekly without waiting list. Secondly, GPs in our regions were encouraged to refer any patient in whom they suspected but doubted about the presence of IA, resulting in a selection of patients in whom there is a lower and more uncertain suspicion of IA than would be the case at one of the more extensively studied Early Arthritis Clinics. The latter are secondary care facilities intended for patients with clear presence or a very high suspicion of IA. Other strengths are the validation of our findings in two cohorts and critical evaluation of the added clinical relevance by adjusting for previously reported diagnostic variables.

A limitation of our study is that data were not collected in primary care practices. Given the relatively low incidence of IA in primary care, it must be noted that prospective research on this subject in general practices would be particularly challenging and resource-intensive. Still, validation in primary care is the next step to be taken. Although the sensitivity and specificity are expected to remain stable across differences in prevalence of IA, positive and negative predictive values are dependent on prior risks and may differ in primary care. Another limitation is that it is unknown how many patients were referred to but did not visit the EA*R*C. Although we assumed this number to be relatively low because of the accessible nature of the EA*R*Cs, it may have influenced the prevalence of IA in the study population. Nevertheless, the prevalence observed in this study suggests that GPs in our region already

For peer review only - http://bmjopen.bmj.com/site/about/guidelines.xhtml

perform quite well in prioritising referrals, since their suspicion of IA was correct in almost half of referred patients. This is in line with previous findings from the EA*R*C.[16,21] Finally, as data were collected cross-sectionally and not longitudinally, analyses were not stratified for final diagnoses and patients who would develop RA were not studied separately. On the other hand, patients with other types of IA (e.g. spondylo-, psoriatic and undifferentiated arthritis) will most likely benefit from early treatment and referral to a rheumatologist as well.[3-5]

In conclusion, we studied the discriminative ability of functional limitations measured by the HAQ and individual HAQ-questions in patients with suspected early IA. One question on difficulties with dressing by itself had a discriminative ability for actual presence of IA that equalled the full HAQ score and was validated in two cohorts. Although further research in primary care settings is necessary, the current data illustrate that a binary response to the question “Are you able to dress yourself, including shoelaces and buttons?” is helpful in the assessment of patients with suspected early IA and could be used in future internet or mobile app based tools aiming for early identification of IA.

For peer review only

For peer review only - http://bmjopen.bmj.com/site/about/guidelines.xhtml

DECLARATONS

Acknowledgements

We thank Xanthe M.E. Matthijssen for her statistical assistance.

Contributorship

BTvD, HWvS and AHMvHM designed the study.

BTvD, HWvS, EB and AHMvHM contributed to data collection.

All authors were involved in interpretation of data.

BTvD, EN and AHMvHM drafted and edited the manuscript.

All authors critically revised the manuscript.

Funding

This work was supported by the European Research Council (ERC) under the European Union's Horizon 2020 research and innovation programme (Starting grant, agreement No. 714312), and the Dutch Arthritis Society.

These organisations were not involved in in the design, execution or reporting of this study.

Competing interests

EB as an employee of the UMCG received speaker fees and consulting fees from Roche in 2017 and 2018 which were paid to the UMCG (outside the submitted work).

Otherwise non declared.

Ethics approval

The Leiden University Medical Centre (LUMC) medical ethical committee approved the study (P16.163) and granted a waiver for obtaining written informed consent in accordance

For peer review only - http://bmjopen.bmj.com/site/about/guidelines.xhtml

with Dutch law on medical research due to data collection being limited to data acquired as part of usual care.

Data sharing statement

Data are available from BTvD (e-mail; B.T.van_Dijk@lumc.nl) upon reasonable request.

For peer review only

REFERENCES

1. Monti S, Montecucco C, Bugatti S, Caporali R. Rheumatoid arthritis treatment: the earlier the better to prevent joint damage. *RMD Open*. 2015;1(Suppl 1):e000057.

2. van der Linden MPM, le Cessie S, Raza K, van der Woude D, Knevel R, Huizinga TWJ, et al. Long-term impact of delay in assessment of patients with early arthritis. *Arthritis & Rheumatism*. 2010;62(12):3537-46.

3. Carron P, Varkas G, Cypers H, Van Praet L, Elewaut D, Van den Bosch F. Anti-TNF-induced remission in very early peripheral spondyloarthritis: the CRESPA study. 2017;76(8):1389-95.

4. Lopez-Olivo MA, Kakpovbia-Eshareturi V, des Bordes JK, Barbo A, Christensen R, Suarez-Almazor ME. Treating Early Undifferentiated Arthritis: A Systematic Review and Meta-Analysis of Direct and Indirect Trial Evidence. *Arthritis care & research*. 2018;70(9):1355-65.

5. McLaughlin M, Ostor A. Early treatment of psoriatic arthritis improves prognosis. *The Practitioner*. 2014;258(1777):21-4, 3.

6. Combe B, Landewe R, Daien CI, Hua C, Aletaha D, Alvaro-Gracia JM, et al. 2016 update of the EULAR recommendations for the management of early arthritis. *Annals of the rheumatic diseases*. 2017;76(6):948-59.

7. Meyfroidt S, Stevens J, De Lepeleire J, Westhovens R, De Cock D, Van der Elst K, et al. A general practice perspective on early rheumatoid arthritis management: A qualitative study from Flanders. *Eur J Gen Pract*. 2015;21(4):231-7.

8. NIVEL. Zorgregistraties eerste lijn: Incidenties en prevalenties [Internet]. 2017. https://www.nivel.nl/nl/zorgregistraties-eerste-lijn/incidenties-en-prevalenties (visited on 5 Feb 2019).

9. Jordan KP, Jöud A, Bergknut C, Croft P, Edwards JJ, Peat G, et al. International comparisons of the consultation prevalence of musculoskeletal conditions using population-based healthcare data from England and Sweden. *Ann Rheum Dis*. 2014;73(1):212-8.

10. Jordan KP, Kadam UT, Hayward R, Porcheret M, Young C, Croft P. Annual consultation prevalence of regional musculoskeletal problems in primary care: an observational study. *BMC Musculoskelet Disord*. 2010;11(1):144.

11. Raza K, Stack R, Kumar K, Filer A, Detert J, Bastian H, et al. Delays in assessment of patients with rheumatoid arthritis: variations across Europe. *Ann Rheum Dis*. 2011;70(10):1822-5.

12. Krebs P, Duncan DT. Health App Use Among US Mobile Phone Owners: A National Survey. *JMIR mHealth uHealth*. 2015;3(4):e101.

13. Subhi Y, Bube SH, Rolskov Bojsen S, Skou Thomsen AS, Konge L. Expert Involvement and Adherence to Medical Evidence in Medical Mobile Phone Apps: A Systematic Review. *JMIR mHealth uHealth*. 2015;3(3):e79.

14. Fries JF, Spitz P, Kraines RG, Holman HR. Measurement of patient outcome in arthritis. *Arthritis Rheum*. 1980;23(2):137-45.

15. Bruce B, Fries JF. The Stanford Health Assessment Questionnaire: dimensions and practical applications. *Health Qual Life Outcomes*. 2003;1:20.

For peer review only - http://bmjopen.bmj.com/site/about/guidelines.xhtml

16. van Nies JA, Brouwer E, van Gaalen FA, Allaart CF, Huizinga TW, Posthumus MD, et al. Improved early identification of arthritis: evaluating the efficacy of Early Arthritis Recognition Clinics. *Annals of the rheumatic diseases*. 2013;72(8):1295-301.

17. Newsum EC, de Waal MWM, van Steenbergen HW, Gussekloo J, van der Helm-van Mil AHM. How do general practitioners identify inflammatory arthritis? A cohort analysis of Dutch general practitioner electronic medical records. *Rheumatology (Oxford)*. 2016;55(5):848-53.

18. Nederlands Huisartsen Genootschap. NHG-Standaard Artritis (eerste herziening) [Internet]. 2017. https://www.nhg.org/standaarden/volledig/nhg-standaard-artritis (visited on 5 Feb 2019).

19. Ahlmen M, Svensson B, Albertsson K, Forslind K, Hafstrom I. Influence of gender on assessments of disease activity and function in early rheumatoid arthritis in relation to radiographic joint damage. *Ann Rheum Dis*. 2010;69(1):230-3.

20. Krishnan E, Sokka T, Hakkinen A, Hubert H, Hannonen P. Normative values for the Health Assessment Questionnaire disability index: benchmarking disability in the general population. *Arthritis Rheum*. 2004;50(3):953-60.

21. ten Brinck RM, van Dijk BT, van Steenbergen HW, le Cessie S, Numans ME, Hider SL, et al. Development and validation of a clinical rule for recognition of early inflammatory arthritis. *BMJ Open*. 2019;8(11):e023552.

22. White IR, Royston P, Wood AM. Multiple imputation using chained equations: Issues and guidance for practice. *Stat Med*. 2011;30(4):377-99.

23. Marshall A, Altman DG, Holder RL, Royston P. Combining estimates of interest in prognostic modelling studies after multiple imputation: current practice and guidelines. *BMC Med Res Methodol*. 2009;9:57.

24. Bruce B, Fries JF. The Stanford Health Assessment Questionnaire: a review of its history, issues, progress, and documentation. *J Rheumatol*. 2003;30(1):167-78.

25. Bell MJ, Tavares R, Guillemin F, Bykerk VP, Tugwell P, Wells GA. Development of a self-administered early inflammatory arthritis detection tool. *BMC Musculoskelet Disord*. 2010;11:50.

26. Khraishi M, Uphall E, Mong J. The self-administered rheumatoid arthritis (RA) screening questionnaire (RASQ) is a simple and effective tool to detect RA patients. *Ann Rheum Dis*. 2010;69 (suppl 3):374.

27. Maksymowych WP, Russell A, Homik J, Keeling S. E-triage Rheumatoid Arthritis Study in Early Arthritis (ERASE) [Internet]. 2007. http://www.arthritisdoctor.ca/erase-intro.php (visited on 28 March 2019).

28. Singh S, Kumar A, Panneerselvam K, Vennila JJ. Diagnosis of arthritis through fuzzy inference system. *J Med Syst*. 2012;36(3):1459-68.

29. Tavares R, Huang S, Bykerk VP, Bell MJ. A parallel group cohort to determine the measurement properties of an early inflammatory arthritis detection tool. *Rheumatology (Oxford)*. 2013;52(11):2077-85.

30. Almoallim H, Janoudi N, Attar SM, Garout M, Algohary S, Siddiqui MI, et al. Determining early referral criteria for patients with suspected inflammatory arthritis presenting to primary care physicians: a cross-sectional study. *Open Access Rheumatol*. 2017;9:81-90.

For peer review only

For peer review only - http://bmjopen.bmj.com/site/about/guidelines.xhtml

31. Croydon Health Services NHS Foundation Trust. Early arthritis services [Internet]. https://www.croydonhealthservices.nhs.uk/a-to-z-of-services/service/early-arthritis-service-151/ (visited on 19 March 2019).

32. Emery P, Breedveld FC, Dougados M, Kalden JR, Schiff MH, Smolen JS. Early referral recommendation for newly diagnosed rheumatoid arthritis: evidence based development of a clinical guide. *Ann Rheum Dis*. 2002;61(4):290-7.

33. Hampshire Hospitals NHS Foundation Trust. Early Arthritis Clinic referral form [Internet]. https://www.hampshirehospitals.nhs.uk/media/616367/updated_eac_form.pdf (visited on 19 March 2019).

34. Hülsemann J, Mattussek S, Siegmund-Schultze E, Zeidler H. [Early diagnosis of arthritis and spondyloarthritis within the framework of integrated health care in Lower Saxony]. *Z Rheumatol*. 2006;65(1):70-4.

35. Musgrove Park Hospital. Early Arthritis Referral Form [Internet]. https://www.tsft.nhs.uk/media/353049/early-arthritis-final.pdf (visited on 19 March 2019).

36. The National Institute for Health and Care Excellence (NICE). Rheumatoid arthritis in adults: management [Internet]. 2018. https://www.nice.org.uk/guidance/ng100 (visited on 19 March 2019).

For peer review only

For peer review only - http://bmjopen.bmj.com/site/about/guidelines.xhtml

TABLES AND FIGURES

Table 1. Characteristics of patients visiting the Early Arthritis *Recognition* Clinics

	Derivation cohort (n = 997)	1st Validation cohort (n = 506)	2nd Validation cohort (n = 557)
**Inclusion period, years**	2010–2014	2010–2014	2015–2018
**Male, n (%)**	290 (29)	177 (35)	183 (33)
**Age in years, mean ± SD**	51 ± 16	52 ± 16	54 ± 16
**Symptom duration in weeks, median (IQR)**	12 (4–62)	18 (5–69)	13 (4–57)
**Acute onset of symptoms, n (%)**	368 (38)	187 (38)	203 (38)
**Morning stiffness in minutes, median (IQR)**	10 (0–30)	10 (0–31)	10 (0–30)
**Number of painful joints, median (IQR)**	7 (3–15)	9 (4–18)	8 (2–17)
**Number of patient-reported swollen joints, median (IQR)**	2 (1–5)	3 (1–8)	2 (1–5)
**Difficulties with making a fist, n (%)**	242 (47)	130 (54)	291 (54)
**Arthralgia location, n (%)**			
≥1 hand or wrist joint	810 (81)	424 (84)	455 (82)
≥1 forefoot joint	254 (25)	142 (28)	152 (27)
≥1 hand, wrist or forefoot joint	869 (87)	454 (90)	486 (87)
Exclusively large joints*	22 (2)	18 (4)	9 (2)
**HAQ-score, mean ± SD**	0.77 ± 0.59	0.88 ± 0.67	–**
**IA present at joint examination, n (%)**	426 (43)	267 (53)	193 (35)

Legend

Original, unimputed data

* Shoulder, elbow, hip, knee or ankle

** Patients in the second validation cohort did not complete the full HAQ but only the question on difficulties with dressing.

Abbreviations: SD = standard deviation; IQR = interquartile range; IA = inflammatory arthritis.

For peer review only - http://bmjopen.bmj.com/site/about/guidelines.xhtml

Table 2. Association between presence of IA and the degree of difficulties with dressing in the derivation cohort (n = 997), with and without dichotomisation into binary scores

Difficulties with dressing		IA n (%*)	No IA n (%*)	OR (95%CI)	AUC (95%CI)
**Categorical****	0	169 (40)	310 (54)	1.00 (ref)	0.58 (0.55–0.62)
	1	188 (44)	210 (37)	1.64 (1.24–2.16)	
	2	62 (15)	45 (8)	2.49 (1.61–3.84)	
	3	7 (2)	6 (1)	2.20 (0.73–6.66)	
**Binary**	no (0)	169 (40)	310 (54)	1.00 (ref)	0.57 (0.54–0.61)
	yes (≥ 1)	257 (60)	261 (46)	1.80 (1.39–2.33)	

Legend

Results were pooled across 30 imputations; percentages and numbers were rounded.

* Percentage of the total number of patients with and without IA, respectively

** 0 = ‘without any difficulty’; 1 = ‘with some difficulty’; 2 = ‘with much difficulty’; 3 = ‘unable to do’

Abbreviations: IA = inflammatory arthritis; OR = odds ratio; CI = confidence interval; AUC = area under the receiver operating characteristics curve; ref = reference.

For peer review only

Table 3. Presence of difficulties with dressing associated with presence of IA independently from other clinical variables.

	OR (95%CI) univariable	OR (95%CI) age, gender adjusted	OR (95%CI) multivariable*
**Derivation cohort (n = 997)**	1.80 (1.39–2.33)	1.83 (1.41–2.39)	1.71 (1.27–2.32)
**1st Validation cohort (n = 506)**	2.00 (1.39–2.87)	1.89 (1.31–2.72)	1.64 (1.08–2.50)
**2nd Validation cohort (n = 557)**	2.14 (1.48–3.10)	1.95 (1.32–2.86)	1.87 (1.20–2.92)

Legend

Results were pooled across 30 imputations.

* Adjusted for: being male, age ≥60 years, symptom duration, acuteness of symptom onset, morning stiffness >60 minutes, number of painful joints, presence of patient-reported swollen joint(s) and difficulties with making a fist (see supplementary Table S2 for details).

Abbreviations: IA = inflammatory arthritis; OR = odds ratio; CI = confidence interval.

For peer review only

For peer review only - http://bmjopen.bmj.com/site/about/guidelines.xhtml

Figure 1. Observed probabilities of IA according to (A) the total HAQ; (B) the degree of difficulties with dressing; and (C) the presence of difficulties with dressing

IMAGE UPLOADED SEPARATELY

Legend

A. Dot sizes vary according to the number of patients with that particular total HAQ-score. The fit line was calculated by means of binary logistic regression.

Abbreviations: IA = Inflammatory arthritis; HAQ = Health Assessment Questionnaire Disability Index.

Figure 2. Test characteristics of the presence of difficulties with dressing (scores ≥1), with IA at physical examination as outcome

IMAGE UPLOADED SEPARATELY

Legend

Results were pooled across 30 imputations.

Error bars indicate the 95% confidence interval.

Abbreviations: IA = Inflammatory arthritis; Sens. = sensitivity; Spec. = specificity; PPV = positive predictive value; NPV = negative predictive value.

For peer review only

For peer review only - http://bmjopen.bmj.com/site/about/guidelines.xhtml

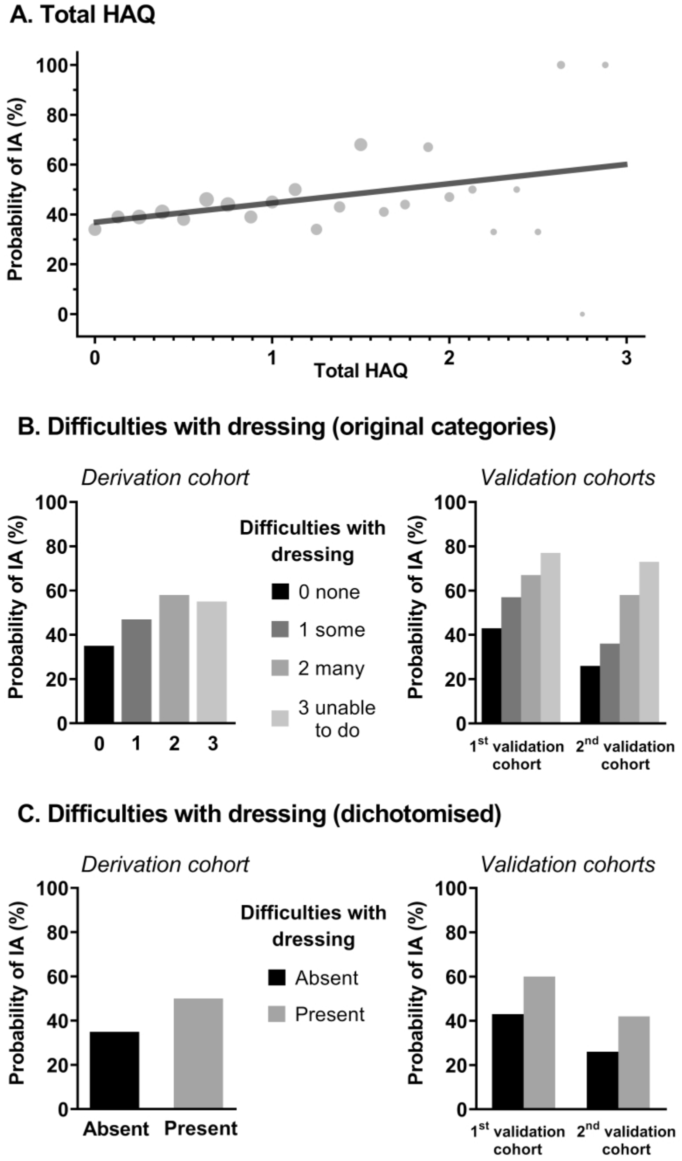

Figure 1. Observed probabilities of IA according to (A) the total HAQ; (B) the degree of difficulties with dressing; and (C) the presence of difficulties with dressing

169x273mm (600 x 600 DPI)

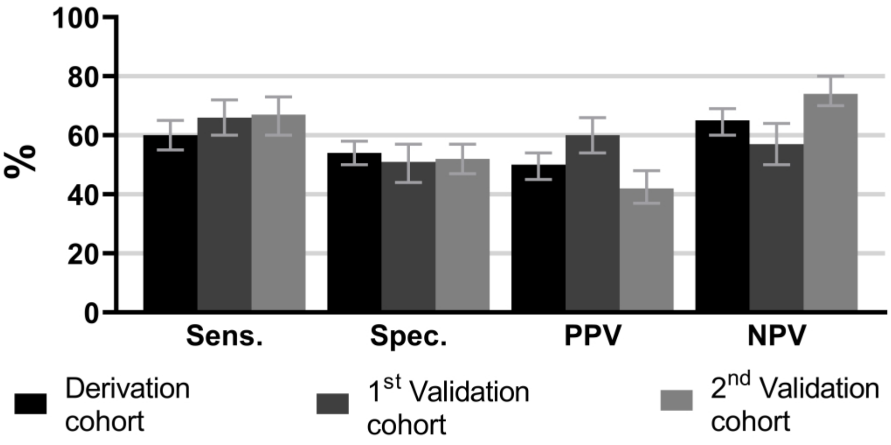

Figure 2. Test characteristics of the presence of difficulties with dressing (scores ≥1), with IA at physical examination as outcome

169x83mm (1200 x 1200 DPI)

Supplementary file

Table S1. Discriminative ability for the presence of IA of the total HAQ-score and its individual questions in the derivation cohort (n = 997)

Category	Item*	AUC (95%CI)	p
–	Total HAQ-score	0.55 (0.52–0.59)	0.006
**(1) Dressing & grooming**	(A) … Dress yourself, including shoelaces and buttons?	0.58 (0.55–0.62)	<0.001
	(B) … Wash your hair?	0.52 (0.48–0.56)	0.301
**(2) Arising**	(A) … Stand up from a straight chair?	0.55 (0.51–0.58)	0.016
	(B) … Get in and out of bed?	0.53 (0.49–0.57)	0.104
**(3) Eating**	(A) … Cut meat?	0.50 (0.46–0.54)	0.897
	(B) … Lift a full cup or glass to your mouth?	0.51 (0.47–0.55)	0.619
	(C) … Open a new milk carton?	0.48 (0.45–0.52)	0.372
**(4) Walking**	(A) … Walk outdoors on flat ground?	0.57 (0.53–0.60)	0.001
	(B) … Climb up five steps?	0.56 (0.52–0.59)	0.003
**(5) Hygiene**	(A) … Wash and dry your body?	0.54 (0.50–0.58)	0.041
	(B) … Get in and out of a tub bath?	0.53 (0.49–0.57)	0.138
	(C) … Get on and off the toilet?	0.56 (0.52–0.59)	0.003
**(6) Reach**	(A) … Reach and get down a 2½kg object (such as a bag of sugar) from above your head?	0.51 (0.47–0.55)	0.567
	(B) … Bend down to pick up clothing from the floor?	0.52 (0.48–0.56)	0.300
**(7) Grip**	(A) … Open car doors?	0.53 (0.49–0.57)	0.145
	(B) … Open previously opened jars?	0.49 (0.45–0.52)	0.464
	(C) … Turn a faucet on and off?	0.51 (0.47–0.55)	0.514
**(8) Activities**	(A) … Run errands and shop?	0.55 (0.51–0.59)	0.007
	(B) … Get in and out of a car?	0.54 (0.50-0.58)	0.037
	(C) … Do chores such as vacuuming or yard work?	0.55 (0.51–0.59)	0.011

Legend

* Names of categories are shown, as well as individual questions (at the EA*R*C, Dutch translations were used). Each question starts with 'Are you able to …'. Patients indicated the degree of difficulties on each question with 'without any difficulty' (0), 'with some difficulty' (1), 'with much difficulty' (2) or 'unable to do' (3).

Abbreviations: IA = inflammatory arthritis; HAQ = Health Assessment Questionnaire Disability Index; AUC = area under the receiver operating characteristics curve; CI = confidence interval.

For peer review only

Table S2. Presence of difficulties with dressing associated with presence of IA independently from other clinical variables* (detailed overview of multivariable model)

		Derivation cohort (n = 997) OR (95%CI)	1st Validation cohort (n = 506) OR (95%CI)	2nd Validation cohort (n = 557) OR (95%CI)
**Male**		1.99 (1.47–2.70)	1.36 (0.91–2.03)	2.47 (1.66–3.68)
**Age ≥ 60 years**		1.82 (1.35–2.45)	1.40 (0.93–2.10)	1.90 (1.28–2.83)
**Symptom duration in weeks**	≥ 52	1.00 (ref)	1.00 (ref)	1.00 (ref)
	0–5.99	3.63 (2.45–5.39)	1.48 (0.86–2.53)	2.39 (1.38–4.17)
	6–51.99	2.16 (1.50–3.13)	1.24 (0.77–1.99)	1.92 (1.13–3.27)
**Acute onset of symptoms**		0.97 (0.72–1.32)	1.05 (0.69–1.58)	1.19 (0.77–1.83)
**Morning stiffness > 60 minutes**		1.34 (0.84–2.15)	1.59 (0.86–2.93)	0.84 (0.45–1.57)
**Number of painful joints**	0	1.00 (ref)	0.00**	1.00 (ref)
	1–3	4.45 (1.36–14.53)	1.54 (0.95–2.50)	2.70 (0.66–11.00)
	≥ 4	2.34 (0.73–7.53)	1.00 (ref)	1.60 (0.40–6.44)
**Patient-reported swollen joint(s) ≥ 1**		1.97 (1.27–3.04)	1.76 (0.95–3.25)	1.74 (1.07–2.81)
**Difficulties with making a fist**		1.31 (0.92–1.88)	1.54 (0.94–2.54)	1.57 (1.01–2.46)
**Difficulties with dressing**	yes (≥ 1)	1.71 (1.27–2.32)	1.64 (1.08–2.50)	1.87 (1.20–2.92)

Legend

Results were pooled across 30 imputations.

* These variables were selected based on a recent study from the Leiden EA*R*C that determined clinical variables that associated with the presence of IA.

** In the Groningen validation cohort none of the patients who indicated no painful joints on the questionnaire were positive for IA upon joint examination. Therefore, no meaningful CI could be calculated for this group and patients with ≥4 painful joints were assigned as reference group instead.

Abbreviations: IA = inflammatory arthritis; OR = odds ratio; CI = confidence interval, ref = reference.

For peer review only

Table S3. Characteristics of patients with and without available data on difficulties with dressing

	Derivation cohort			1st Validation cohort			2nd Validation cohort		
**Data on difficulties with dressing:**	**Available (n = 956)**	**Missing (n = 41)**	**p***	**Available (n = 495)**	**Missing (n = 11)**	**p***	**Available (n = 542)**	**Missing (n = 15)**	**p***
**Male, n (%)**	275 (29)	15 (37)	0.294	174 (35)	3 (27)	0.755	180 (33)	3 (20)	0.406
**Age in years, mean ± SD**	51 ± 16	57 ± 17	0.015	51 ± 16	57 ± 10	0.237	54 ± 16	58 ± 12	0.312
**Symptom duration in weeks, median (IQR)**	13 (4–62)	9 (3–72)	0.792	18 (5–69)	7 (5–64)	0.519	13 (4–59)	13 (5–24)	0.750
**Acute onset of symptoms, n (%)**	353 (38)	15 (41)	0.731	185 (38)	2 (20)	0.333	201 (37)	2 (29)	0.713
**Morning stiffness in minutes, median (IQR)**	10 (0–30)	4 (0–60)	0.983	10 (0–33)	10 (1–190)	0.776	10 (0–30)	0 (0–1)	0.012
**Number of painful joints, median (IQR)**	7 (3–14)	6 (2–25)	0.941	9 (4–18)	9 (3–20)	0.840	8 (3–17)	1 (1–9)	0.021
**Number of patient-reported swollen joints, median (IQR)**	2 (1–5)	2 (0–5)	0.503	3 (1–8)	5 (2–8)	0.698	2 (1–5)	1 (0–5)	0.317
**Difficulties with making a fist, n (%)**	239 (48)	3 (23)	0.095	128 (54)	2 (50)	1.000	288 (55)	3 (50)	1.000

Legend

Percentages are based on available questionnaire entries.

* Unpaired t-tests, Fisher's Exact tests and Mann-Whitney U tests were used as appropriate.

Abbreviations: SD = standard deviation; IQR = interquartile range.

For peer review only

Table S4. (sensitivity analyses limited to unimputed data) Association between presence of difficulties with dressing and presence of IA in the derivation and validation cohorts

	OR (95%CI) univariable	OR (95%CI) age, gender adjusted	OR (95%CI) multivariable*
**Derivation cohort**	1.83 (1.41–2.38)	1.87 (1.43–2.44)	1.59 (0.98–2.57)
**1st Validation cohort**	2.02 (1.41–2.91)	1.92 (1.33–2.77)	1.70 (0.83–3.84)
**2nd Validation cohort**	2.19 (1.51–3.17)	1.99 (1.36–2.93)	1.91 (1.16–3.16)

Legend

* Point estimates are similar to those obtained in the main analyses, but have broader confidence intervals since only complete cases could be analysed. Covariates: being male, age ≥60 years, symptom duration, acuteness of symptom onset, morning stiffness >60 minutes, number of painful joints, presence of patient-reported swollen joint(s) and difficulties with making a fist.

Abbreviations: IA = inflammatory arthritis; OR = odds ratio; CI = confidence interval.

For peer review only

STROBE 2007 (v4) Statement—Checklist of items that should be included in reports of *cross-sectional studies*

Section/Topic	Item #	Recommendation	Reported on page #
**Title and abstract**	1	(*a*) Indicate the study's design with a commonly used term in the title or the abstract	1, 2
		(*b*) Provide in the abstract an informative and balanced summary of what was done and what was found	2
**Introduction**			
Background/rationale	2	Explain the scientific background and rationale for the investigation being reported	5, 6
Objectives	3	State specific objectives, including any prespecified hypotheses	6
**Methods**			
Study design	4	Present key elements of study design early in the paper	6–8
Setting	5	Describe the setting, locations, and relevant dates, including periods of recruitment, exposure, follow-up, and data collection	6–8
Participants	6	(*a*) Give the eligibility criteria, and the sources and methods of selection of participants	6, 7
Variables	7	Clearly define all outcomes, exposures, predictors, potential confounders, and effect modifiers. Give diagnostic criteria, if applicable	8, 9
Data sources/ measurement	8*	For each variable of interest, give sources of data and details of methods of assessment (measurement). Describe comparability of assessment methods if there is more than one group	7
Bias	9	Describe any efforts to address potential sources of bias	9, 10
Study size	10	Explain how the study size was arrived at	7
Quantitative variables	11	Explain how quantitative variables were handled in the analyses. If applicable, describe which groupings were chosen and why	8, 9
Statistical methods	12	(*a*) Describe all statistical methods, including those used to control for confounding	9, 10
		(*b*) Describe any methods used to examine subgroups and interactions	*NA*
		(*c*) Explain how missing data were addressed	9, 10
		(*d*) If applicable, describe analytical methods taking account of sampling strategy	*NA*
		(*e*) Describe any sensitivity analyses	10
**Results**			

For peer review only

Participants	13*	(a) Report numbers of individuals at each stage of study—eg numbers potentially eligible, examined for eligibility, confirmed eligible, included in the study, completing follow-up, and analysed	21
		(b) Give reasons for non-participation at each stage	7
		(c) Consider use of a flow diagram	*NA*
Descriptive data	14*	(a) Give characteristics of study participants (eg demographic, clinical, social) and information on exposures and potential confounders	21
		(b) Indicate number of participants with missing data for each variable of interest	9
Outcome data	15*	Report numbers of outcome events or summary measures	10–12, 21–23
Main results	16	(*a*) Give unadjusted estimates and, if applicable, confounder-adjusted estimates and their precision (eg, 95% confidence interval). Make clear which confounders were adjusted for and why they were included	23
		(*b*) Report category boundaries when continuous variables were categorized	22
		(*c*) If relevant, consider translating estimates of relative risk into absolute risk for a meaningful time period	*NA*
Other analyses	17	Report other analyses done—eg analyses of subgroups and interactions, and sensitivity analyses	12
**Discussion**			
Key results	18	Summarise key results with reference to study objectives	13
Limitations	19	Discuss limitations of the study, taking into account sources of potential bias or imprecision. Discuss both direction and magnitude of any potential bias	15
Interpretation	20	Give a cautious overall interpretation of results considering objectives, limitations, multiplicity of analyses, results from similar studies, and other relevant evidence	16
Generalisability	21	Discuss the generalisability (external validity) of the study results	15, 16
**Other information**			
Funding	22	Give the source of funding and the role of the funders for the present study and, if applicable, for the original study on which the present article is based	17

*Give information separately for cases and controls in case-control studies and, if applicable, for exposed and unexposed groups in cohort and cross-sectional studies.

Note: An Explanation and Elaboration article discusses each checklist item and gives methodological background and published examples of transparent reporting. The STROBE checklist is best used in conjunction with this article (freely available on the Web sites of PLoS Medicine at http://www.plosmedicine.org/, Annals of Internal Medicine at http://www.annals.org/, and Epidemiology at http://www.epidem.com/). Information on the STROBE Initiative is available at www.strobe-statement.org.

For peer review only

Section & Topic	No	Item	Reported on page #
**TITLE OR ABSTRACT**			
	**1**	Identification as a study of diagnostic accuracy using at least one measure of accuracy (such as sensitivity, specificity, predictive values, or AUC)	1–2
**ABSTRACT**			
	**2**	Structured summary of study design, methods, results, and conclusions (for specific guidance, see STARD for Abstracts)	2
**INTRODUCTION**			
	**3**	Scientific and clinical background, including the intended use and clinical role of the index test	5–6
	**4**	Study objectives and hypotheses	6
**METHODS**			
Study design	**5**	Whether data collection was planned before the index test and reference standard were performed (prospective study) or after (retrospective study)	*NA (differed per cohort; see p. 8 under 'HAQ')*
Participants	**6**	Eligibility criteria	6–7
	**7**	On what basis potentially eligible participants were identified (such as symptoms, results from previous tests, inclusion in registry)	6–7
	**8**	Where and when potentially eligible participants were identified (setting, location and dates)	6–7
	**9**	Whether participants formed a consecutive, random or convenience series	2, 7
Test methods	**10a**	Index test, in sufficient detail to allow replication	8
	**10b**	Reference standard, in sufficient detail to allow replication	8
	**11**	Rationale for choosing the reference standard (if alternatives exist)	8
	**12a**	Definition of and rationale for test positivity cut-offs or result categories of the index test, distinguishing pre-specified from exploratory	9
	**12b**	Definition of and rationale for test positivity cut-offs or result categories of the reference standard, distinguishing pre-specified from exploratory	*NA*
	**13a**	Whether clinical information and reference standard results were available to the performers/readers of the index test	8
	**13b**	Whether clinical information and index test results were available to the assessors of the reference standard	8
Analysis	**14**	Methods for estimating or comparing measures of diagnostic accuracy	9
	**15**	How indeterminate index test or reference standard results were handled	*NA*
	**16**	How missing data on the index test and reference standard were handled	9–10
	**17**	Any analyses of variability in diagnostic accuracy, distinguishing pre-specified from exploratory	15
	**18**	Intended sample size and how it was determined	6–7
**RESULTS**			
Participants	**19**	Flow of participants, using a diagram	8 (diagram *NA* because of simplicity)
	**20**	Baseline demographic and clinical characteristics of participants	10, 22
	**21a**	Distribution of severity of disease in those with the target condition	*NA* (see p. 8/16)
	**21b**	Distribution of alternative diagnoses in those without the target condition	*NA* (see p. 8/16)
	**22**	Time interval and any clinical interventions between index test and reference standard	*NA* (same visit)
Test results	**23**	Cross tabulation of the index test results (or their distribution) by the results of the reference standard	22
	**24**	Estimates of diagnostic accuracy and their precision (such as 95% confidence intervals)	10–12
	**25**	Any adverse events from performing the index test or the reference standard	*NA*
**DISCUSSION**			
	**26**	Study limitations, including sources of potential bias, statistical uncertainty, and generalisability	15–16
	**27**	Implications for practice, including the intended use and clinical role of the index test	16
**OTHER INFORMATION**			
	**28**	Registration number and name of registry	*NA*
	**29**	Where the full study protocol can be accessed	*NA*

For peer review only - http://bmjopen.bmj.com/site/about/guidelines.xhtml

cc BY SA

	**30**	Sources of funding and other support; role of funders	17

For peer review only

STARD 2015

AIM

STARD stands for "Standards for Reporting Diagnostic accuracy studies". This list of items was developed to contribute to the completeness and transparency of reporting of diagnostic accuracy studies. Authors can use the list to write informative study reports. Editors and peer-reviewers can use it to evaluate whether the information has been included in manuscripts submitted for publication.

EXPLANATION

A **diagnostic accuracy study** evaluates the ability of one or more medical tests to correctly classify study participants as having a **target condition.** This can be a disease, a disease stage, response or benefit from therapy, or an event or condition in the future. A medical test can be an imaging procedure, a laboratory test, elements from history and physical examination, a combination of these, or any other method for collecting information about the current health status of a patient.

The test whose accuracy is evaluated is called **index test.** A study can evaluate the accuracy of one or more index tests. Evaluating the ability of a medical test to correctly classify patients is typically done by comparing the distribution of the index test results with those of the **reference standard**. The reference standard is the best available method for establishing the presence or absence of the target condition. An accuracy study can rely on one or more reference standards.

If test results are categorized as either positive or negative, the cross tabulation of the index test results against those of the reference standard can be used to estimate the **sensitivity** of the index test (the proportion of participants *with* the target condition who have a positive index test), and its **specificity** (the proportion *without* the target condition who have a negative index test). From this cross tabulation (sometimes referred to as the contingency or "2x2" table), several other accuracy statistics can be estimated, such as the positive and negative **predictive values** of the test. Confidence intervals around estimates of accuracy can then be calculated to quantify the statistical **precision** of the measurements.

If the index test results can take more than two values, categorization of test results as positive or negative requires a **test positivity cut-off**. When multiple such cut-offs can be defined, authors can report a receiver operating characteristic (ROC) curve which graphically represents the combination of sensitivity and specificity for each possible test positivity cut-off. The **area under the ROC curve** informs in a single numerical value about the overall diagnostic accuracy of the index test.

The **intended use** of a medical test can be diagnosis, screening, staging, monitoring, surveillance, prediction or prognosis. The **clinical role** of a test explains its position relative to existing tests in the clinical pathway. A replacement test, for example, replaces an existing test. A triage test is used before an existing test; an add-on test is used after an existing test.

Besides diagnostic accuracy, several other outcomes and statistics may be relevant in the evaluation of medical tests. Medical tests can also be used to classify patients for purposes other than diagnosis, such as staging or prognosis. The STARD list was not explicitly developed for these other outcomes, statistics, and study types, although most STARD items would still apply.

DEVELOPMENT

This STARD list was released in 2015. The 30 items were identified by an international expert group of methodologists, researchers, and editors. The guiding principle in the development of STARD was to select items that, when reported, would help readers to judge the potential for bias in the study, to appraise the applicability of the study findings and the validity of conclusions and recommendations. The list represents an update of the first version, which was published in 2003.

More information can be found on http://www.equator-network.org/reporting-guidelines/stard.

For peer review only - http://bmjopen.bmj.com/site/about/guidelines.xhtml
